# Atlantic salmon and sea trout display synchronised smolt migration relative to linked environmental cues

**DOI:** 10.1038/s41598-020-60588-0

**Published:** 2020-02-26

**Authors:** Alison C. Harvey, Kevin A. Glover, Vidar Wennevik, Øystein Skaala

**Affiliations:** 1 Institute of Marine Research (IMR), Bergen, Norway; 2 Institute of Biology, University of Bergen, Bergen, Norway

**Keywords:** Animal migration, Behavioural ecology, Conservation biology

## Abstract

Anadromous salmon and sea trout smolts face challenging migrations from freshwater to the marine environment characterised by high mortality. Therefore, the timing of smolt migration is likely to be critical for survival. Time-series comparing migration of Atlantic salmon and sea trout smolts in the same river, and their response to the same environmental cues, are scarce. Here, we analysed migration timing of ~41 000 Atlantic salmon and sea trout smolts over a 19-year period from the river Guddalselva, western Norway. Trout displayed a longer migration window in earlier years, which decreased over time to become more similar to the salmon migration window. On average, salmon migrated out of the river earlier than trout. Migration of both species was significantly influenced by river water temperature and water discharge, but their relative influence varied across the years. On average, body-length of smolts of both species overlapped, however, size differences were observed within the migration period and among the years. We conclude that salmon and trout smolts in this river are highly synchronised and migrate in response to the same range of linked environmental cues.

## Introduction

The life cycle of an anadromous fish involves migrations between freshwater and marine environments, enabling them to maximise individual fitness by exploiting different habitats^1^. Atlantic salmon (*Salmo salar*) and sea trout (anadromous brown trout; *Salmo trutta*) occur in sympatric anadromous forms throughout most of their range^2^. Both species reproduce in freshwater, and juveniles remain in the river for between 1–8 years before migrating into the marine environment to feed^3^. After a time at sea, adults return to their native river to spawn. Atlantic salmon typically spend between 1–3 years in the ocean, while sea trout can switch between the marine and freshwater environment multiple times over the periods of months to years^3,4^.

Migration to the ocean is a challenging phase in a salmonid’s life cycle: they must undergo a physical transformation known as smoltification to adapt to increased salinity, switch between different food types, and are exposed to novel predators^5^. These challenges interact together, with the result that the migration phase is characterised by high mortality. In general, smolt migration occurs between April and July, depending on temperature and latitude^2^. Day length is believed to be the proximate cue for parr to initiate smoltification in preparation for migration in both Atlantic salmon^6,7^ and brown trout^8^, while the timing of migration is mitigated by other environmental factors^1,9^.

There is evidence for a genetic component to the variation in smolt migration timing, potentially reflecting local adaptions to native environmental conditions^4,10,11^. Genetic differences among populations in smolt migration timing are probably the result of selection on optimal growth and survival opportunities in the marine environment^12–14^. Population differences may therefore be adaptive and may significantly affect survival during seaward migration and subsequent population recruitment. As there is often variability of migration timing within a population, it is likely that the genetic differences in this trait between populations are also mitigated by environmental cues which act to initiate migration, probably through the control of the developmental processes involved in smoltification^9,15,16^. Furthermore, trout and salmon, with their different marine migratory patterns and habitats, may have different optimal timings to enter the marine environment, and may therefore respond differently to environmental cues. The environmental cues which govern migration are closely linked to climate, and changes in environmental parameters due to climate change may thus cause unknown or unwanted changes in migration patterns. Several studies in the marine and freshwater environment have highlighted changes in life history of salmonids associated with climate change^17–19^. It is therefore important to understand the environmental drivers governing differences in patterns and timing of migration among anadromous species and rivers to ensure optimal management strategies and to be able to predict potential impacts from climate change.

Several short- and long-term studies have investigated the influence of environmental variables in triggering migration to the marine environment in Atlantic salmon^15,20^ and sea trout^21,22^. Water temperature and discharge are the two most important environmental variables influencing smolt migration timing in Atlantic salmon^5,15,23^ and sea trout^22,24^. However, most studies have focussed on one species within a river system [although see 2,25], and there is a lack of long-term studies which investigate the influence of environmental variables on the timing of smolt migration in sympatric species simultaneously.

**Figure 1:**
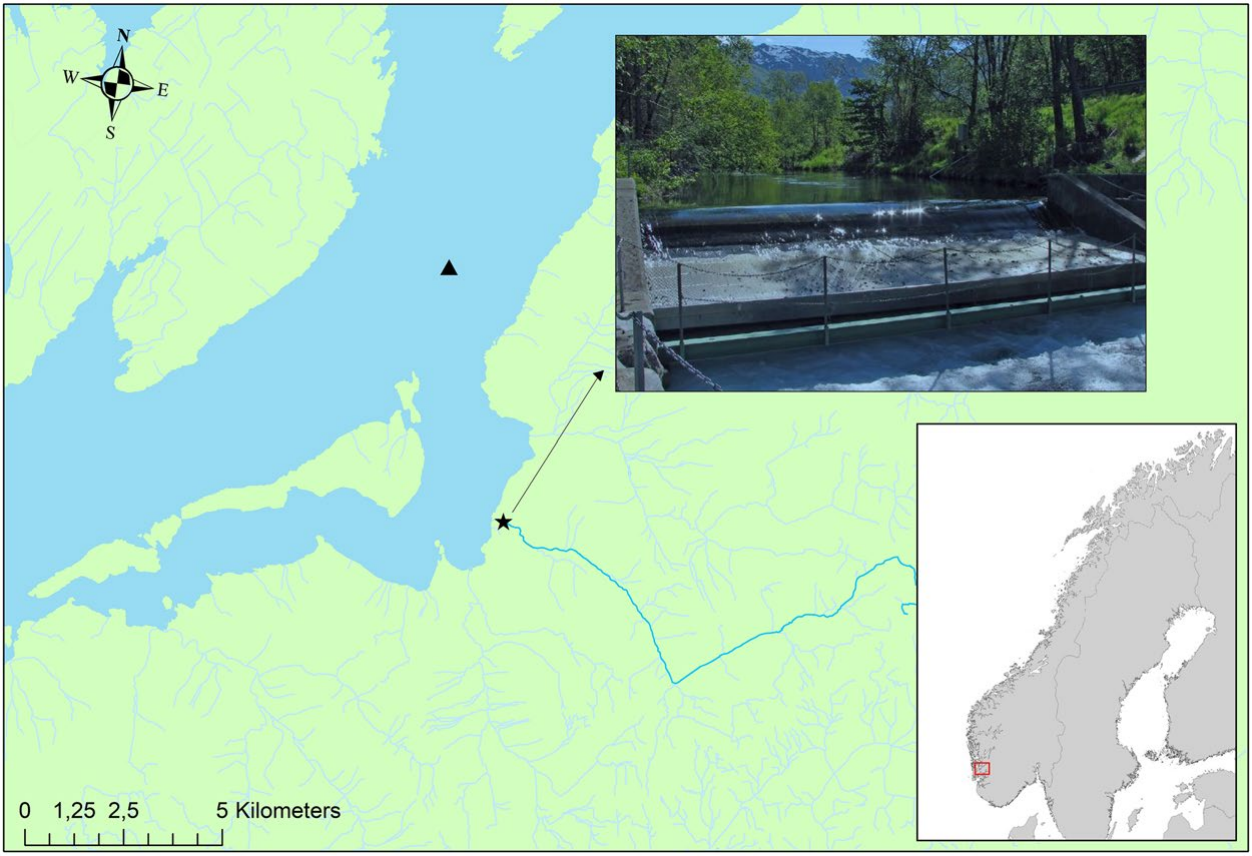
Map of location of Guddalselva showing the location of the Wolf trap, river temperature and discharge (★) and sea temperature (▲) measurement stations. Photo: Øystein Skaala.

The river Guddalselva, western Norway (Fig. 1), includes a trapping facility that has permitted the capture and sampling of the vast majority of the salmon and sea trout smolts leaving the river since 2001. In addition to individual data comprised of ~41 000 anadromous salmon and trout smolts, daily water-temperature and discharge data are available for the river through much of this period. This data set thus provides a rare opportunity to investigate the daily and yearly patterns of salmon and sea trout smolt migration simultaneously over nearly two decades. The overall aim of this study was to investigate temporal variations in smolt migrations. Specifically, to (1) compare migration patterns between sea trout and salmon smolts, (2) investigate the inter-annual variation in peak smolt migration timing, and 3) look for environmental triggers associated with smolt migration peaks.

## Results

### The data

In total, 21 783 salmon and 19 272 sea trout were sampled in the period from 2001 to 2019 (Table 1). Eleven continuous years of data (2009–2019) were available with river temperature. During this period, 17 589 salmon and 10 474 trout smolts migrated. During the period (2001, 2003–2005, 2007–2019) in which sea water temperature and water discharge data were available, a total of 20 932 salmon and 17 283 trout smolts migrated. Throughout the study period (April – June each year), river water temperature ranged from 1.87 °C to 12.43 °C with an average of 5.9 °C, water discharge ranged from 0.16 m^3^/s to 16.86 m^3^/s with an average flow of 4.76 m^3^/s, while sea water temperature ranged from 1.97 °C to 19.41 °C with an average of 9.88 °C. Over the entire study period the length of salmon smolts ranged from 4.7 to 23.8 cm (average 14.07 cm ± 1.13 cm) and the length of trout ranged from 4.3 to 32.2 cm (average 14.85 cm ± 1.80 cm).

**Table 1:** Yearly summary data for the environmental variables, size and numbers of salmon and trout.

Year	Migration window				River	Sea water	River	No. of salmon	No. of trout	Salmon length	Trout length
	Salmon		Trout		temperature	temperature	discharge				
	Start	End	Start	End	range (°C)	range (°C)	range (m/s^3^)				
2001	15. apr	29.mai	15.apr	30.jun	2.01–10.56	5.16–12.40	0.96–13.05	125	1152	14.75 ± 0.78	14.95 ± 1.65
2002	22.apr	27.mai	04.apr	05.jul		4.18–13.48		32	1128	16.41 ± 1.61	14.94 ± 2.09
2003	26.apr	29.mai	18.apr	19.jun		4.54–12.21	1.06–13.55	152	1650	14.53 ± 1.1	14.2 ± 1.62
2004	17.apr	24.mai	17.apr	10.jun		5.71–11.59	1.41–11.86	236	1283	15.53 ± 1.1	14.83 ± 1.54
2005	21.apr	04.jul	03.mai	04.jul		5.20–13.04	2.58–11.32	181	868	13.27 ± 1.28	14.6 ± 1.91
2006	16.apr	18.jun	16.apr	06.jul		6.07–13.79		829	861	13.5 ± 1.01	15.02 ± 1.48
2007	13.apr	25.mai	13.apr	06.jul	2.91–11.19	5.63–14.41	1.41–10.31	1167	1077	14.2 ± 1.07	15.48 ± 1.83
2008	02.apr	09.jun	02.apr	11.jun		3.46–18.12	0.86–14.11	1482	779	14.05 ± 1.08	14.77 ± 1.58
2009	01.apr	18.jun	06.apr	20.jul	4.97–12.43	6.43–15.67	2.79–9.02	353	1075	15.28 ± 0.89	14.74 ± 1.62
2010	19.apr	16.jun	19.apr	09.jul	3.3–10.24	6.48–14.28	0.77–7.17	802	969	14.75 ± 0.98	15.27 ± 1.47
2011	15.apr	17.jun	15.apr	17.jun	2.81–9.78	5.76–13.33	2.71–16.86	1525	635	14.79 ± 0.96	14.78 ± 2.02
2012	12.apr	22.jun	12.apr	22.jun	2.82–8.41	6.59–14.50	1.32–12.33	653	1194	14.82 ± 1.03	14.54 ± 2.08
2013	24.apr	24.jun	24.apr	24.jun	1.87–9.63	6.67–15.33	0.16–11.55	927	496	13.69 ± 0.96	14.6 ± 1.66
2014	31.mar	24.jun	01.apr	24.jun	3.46–9.02	5.39–17.04	2.25–11.64	2456	1164	14.1 ± 1.07	15.52 ± 1.96
2015	22.apr	23.jun	22.apr	23.jun	2.8–7.41	7.72–14.02	1.14–14.03	2316	818	13.6 ± 1.05	15.15 ± 2.06
2016	22.apr	29.jun	22.apr	29.jun	2.57–10.82	7.57–17.70	1.07–11.05	1980	991	13.64 ± 1.06	14.83 ± 1.75
2017	07.apr	19.jun	07.apr	03.jul	3.26–8.82	6.57–15.8	1.81–16.11	3436	969	13.95 ± 1.03	15.29 ± 2.13
2018	16.apr	15.jun	14.apr	18.jun	2.35–10.52	3.83–19.41	0.46–10.04	1628	629	13.90 ± 1.13	14.06 ± 2.11
2019	26.apr	11.jun	26.apr	11.jun	3.56–9.63	4.61–15.28	1.5–7.23	1513	1534	14.23 ± 1.04	14.4 ± 1.72

DoY: Day of the year; se: standard error.

### General migration

**Figure 2:**
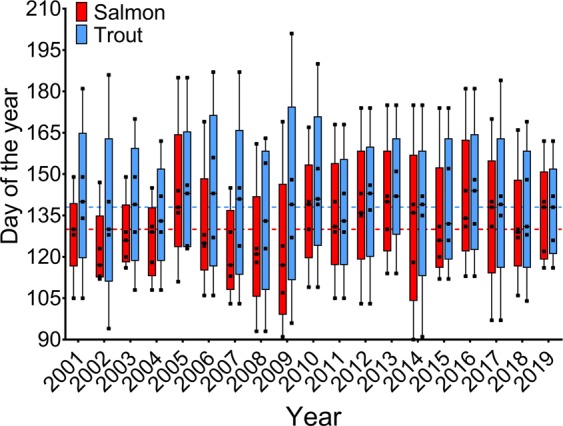
The smolt migration window as days of the year per year for each species. The whiskers represent the start and end day of each migration period, the squares within the boxes represent the 25, 50 and 75% of the total migrating population for Atlantic salmon and sea trout across the entire period. Dotted lines represent the average 50% day of migration across the years for salmon (red line) and trout (blue line).

**Figure 3:**
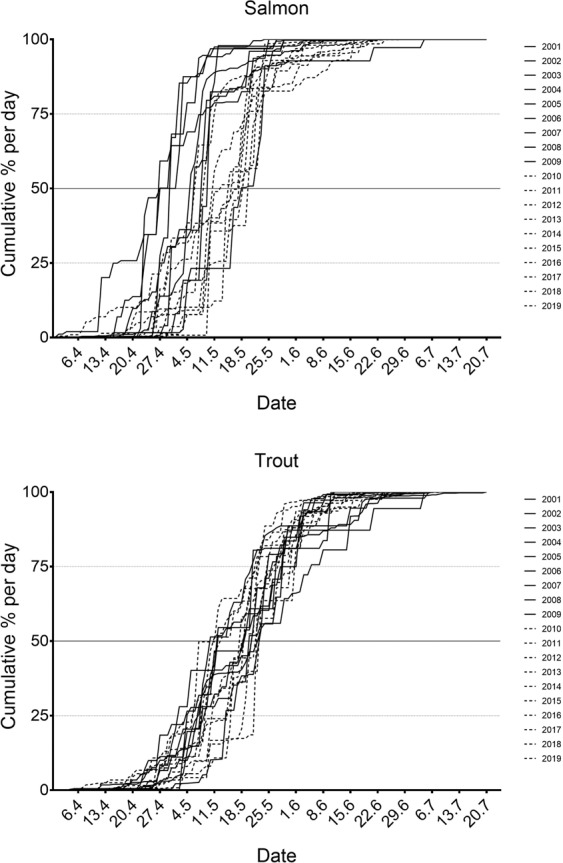
Cumulative migration of Atlantic salmon and sea trout migrating from the river per year and per day over the total study period (2001–2019).

The migration of both species generally occurred in the period between mid to late April and early June. In the early years (2001–2010), the smolt migration window was longer for trout than for salmon. However, in later years (2011–2019), the trout migration window decreased over time, and the migration of both species became more synchronised (Table 1, Fig. 2), and the variation in migration windows for both species across the years was similar (2 weeks). Over all the years, the smolt migration window lasted between 7 and 15 weeks (average: 10 weeks) for trout and 5 and 12 weeks (average: 8 weeks) for salmon (Table 1, Figs. 2 and 3).

**Figure 4:**
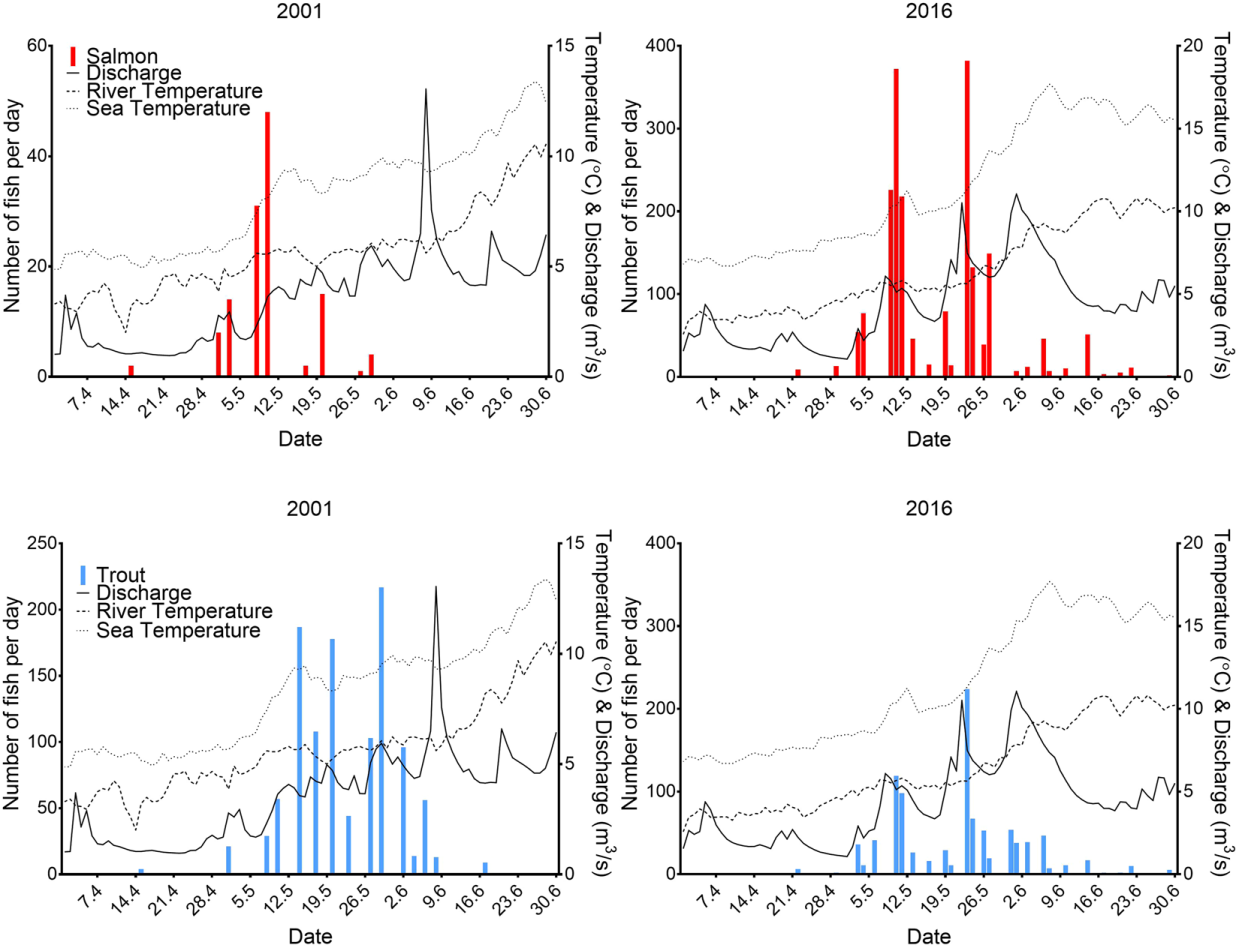
A selection of some years illustrating the daily numbers of Atlantic salmon and sea trout migrating from the river. Daily river water temperature (dashed line), daily sea water temperature (stippled line) (°C) and daily water discharge (solid line) (m^3^/s) are also shown. All years are shown in Supplementary Fig. S1.

The salmon migration tended to peak earlier than trout, with an overall average of 8 days difference between the 50% cumulative migration dates of the species (Figs. 2 and 3). The difference varied over time (between 1 and 24 days) and in later years (after 2010) the difference in the 50% migration date between the species decreased, with an overlap in migration peaks between the species (Fig. 4). Although there was more variation in the 50% cumulative migration dates of the salmon among the years than for trout, with an average difference of 4.5 days between years for trout compared to 7.6 days for salmon. In some years, the migration of both species exhibited more than one peak, and generally, two peaks were visible for both species in the same year (Fig. 4, Supplementary Fig. S1).

### Influence of environmental parameters on daily migration

**Figure 5:**
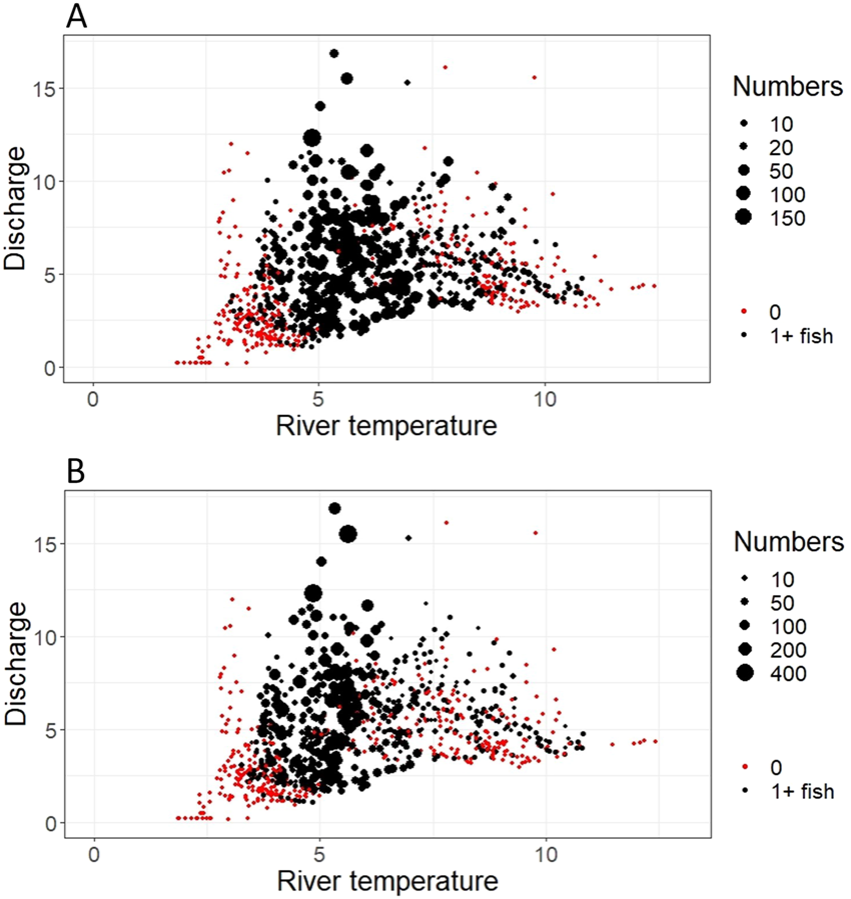
The number of salmon (top) and trout (bottom) migrating out of the river per year in relation to river water temperature (°C) and water discharge (m^3^/s). Red dots indicate days where no fish were observed, black dots indicate days where one or more fish was migrating out the river. The size of the dots is an indication of the number of fish migrating that day, where larger dots indicate higher numbers of fish. All years are shown in Supplementary Fig. S2 (salmon) and S3 (trout).

For both salmon and trout, neither the change in water discharge nor its interaction with year significantly influenced daily migration (Tables 2 and 3). The two-way interactions between river temperature and water discharge with year were significant for both species (Tables 2A and 3A), and the two-way interaction between river temperature and discharge was also significant for both species (Tables 2A and 3A). Therefore, migration was significantly influenced by both environmental parameters, with their influence varying depending on both the year and the interaction between said parameters. For both species, the daily number of fish migrating out of the river was highest at a river temperature of 5 °C across a large range of water discharge values (Fig. 5). At moderate river temperatures (5–8 °C), larger numbers of migrating fish were observed when the river discharge was above 5 m^3^/s (Fig. 5, although this was not always the case, Supplementary Figs. S2 and S3). Generally, in years when both water temperature and water discharge were low (<3 °C and <2 m^3^/s) or river temperature was high (>8 °C) the number of migrating fish was often low or zero (Fig. 5, Supplementary Figs. S2 and S3). In certain years (for example, 2011 Fig. 6, Supplementary Figs. S4 and S5), the daily number of fish migrating out of the river was highest at a river temperature of or above 8 °C. In other years (for example, 2016 Fig. 6, Supplementary Figs. S4 and S5), while the highest numbers of daily counts of fish were observed once again around 5 °C, fish were also recorded migrating out of the river in lower numbers above 8 °C.

**Table 2:** ANOVA output from the generalised linear models investigating the factors affecting daily salmon migration. A: Model 1.1; B: Model 1.2.

*Model terms*	*Chi Square*	*df*	*P value*
**A 1.1**			
Change in discharge	0.88	1	0.35
Year	16.58	10	0.08
**River temperature**	7.61	1	0.01
**Discharge**	82.01	1	0.00
Change in discharge x Year	8.08	10	0.62
**River temperature x Discharge**	21.49	10	0.02
**River temperature x Year**	26.65	10	0.00
**Discharge x Year**	12.55	1	0.00
**B 1.2**			
Change in discharge	2.47	1	0.12
**Year**	36.92	16	0.00
**Discharge**	116.13	1	0.00
**Sea temperature**	5.61	1	0.02
Change in discharge x Year	23.60	16	0.10
**Discharge x Year**	84.11	16	0.00
**Sea temperature x Year**	37.39	16	0.00

Significant terms are shown in bold, df; degrees of freedom, Chi.sq; Chi-square value.

**Table 3:** ANOVA output from the generalised linear models investigating the factors affecting daily trout migration. A: Model 1.1; B: Model 1.2.

*Model terms*	*Chi Square*	*df*	*P value*
**A 1.1**			
Change in discharge	0.24	1	0.62
Year	12.98	10	0.22
**River temperature**	19.62	1	0.00
**Discharge**	40.17	1	0.00
Change in discharge x Year	18.12	10	0.05
**River temperature x Discharge**	23.72	1	0.00
**River temperature x Year**	20.92	10	0.02
**Discharge x Year**	41.37	10	0.00
**B 1.2**			
Change in discharge	2.69	1	0.10
Year	9.48	16	0.89
Sea temperature	0.50	1	0.48
**Discharge**	73.79	1	0.00
Change in discharge x Year	23.61	16	0.10
**Sea temperature x Year**	29.39	16	0.02
**Discharge x Year**	42.32	16	0.00

Significant variables are shown in bold, df; degrees of freedom, Chi.sq; Chi-square value.

**Figure 6:**
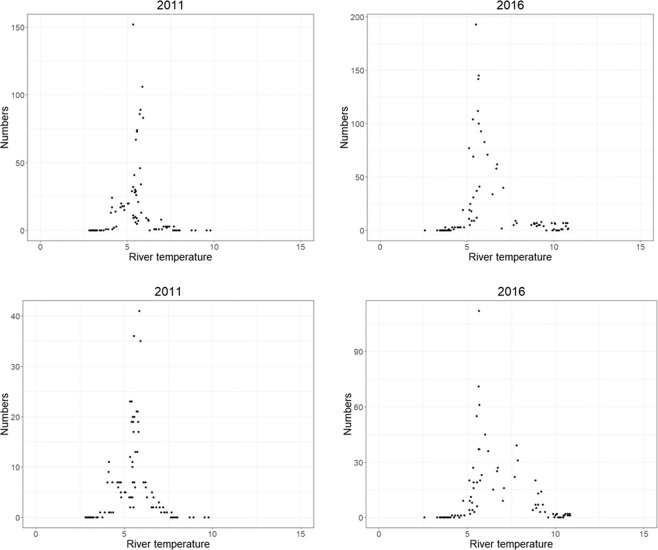
A selection of some years illustrating the number of salmon (top) and trout (bottom) migrating out of the river per year in relation to river temperature (°C). All years are shown in Supplementary Fig. S4 (salmon) and S5 (trout).

**Figure 7:**
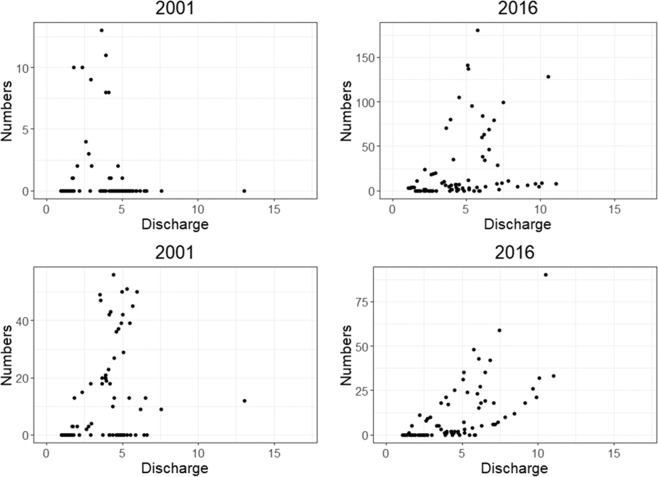
A selection of some years illustrating the number of salmon (top) and trout (bottom) migrating out of the river per year in relation to water discharge (m^3^/s). All years are shown in Supplementary Fig. S6 (salmon) and S7 (trout).

**Figure 8:**
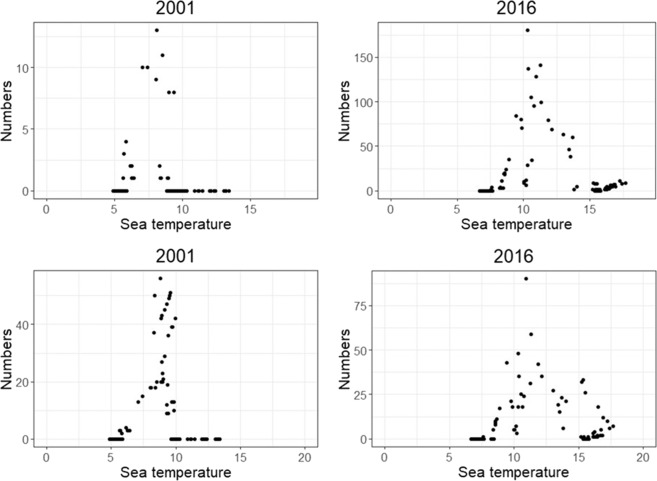
A selection of some years illustrating the number of salmon (top) and trout (bottom) migrating out of the river per year in relation to sea water temperature (°C) outside the river Guddalselva. All years are shown in Supplementary Fig. S8 (salmon) and S9 (trout).

For both species the two-way interactions of sea water temperature and year, and discharge and year, significantly influenced daily migration (Tables 2B and 3B). In both salmon and trout, numbers of fish migrating out the river increased as discharge increased, with peak numbers occurring either at 5 m^3^/s or between 5 and 10 m^3^/s (Fig. 7, Supplementary Figs. S6 and S7). For salmon, between 2001 and 2009, most fish migrated out of the river as the sea temperature in the area was between 5 °C and 10 °C, and from 2010 the peak migration occurred when the nearby sea water temperature was around 10 °C (Fig. 8, Supplementary Figs. S8 and S9). For trout in most years, the peak migration occurred when the sea water temperature in the area was above 10 °C (Fig. 8, Supplementary Figs. S8 and S9).

### Body length of salmonids leaving the river

**Figure 9:**
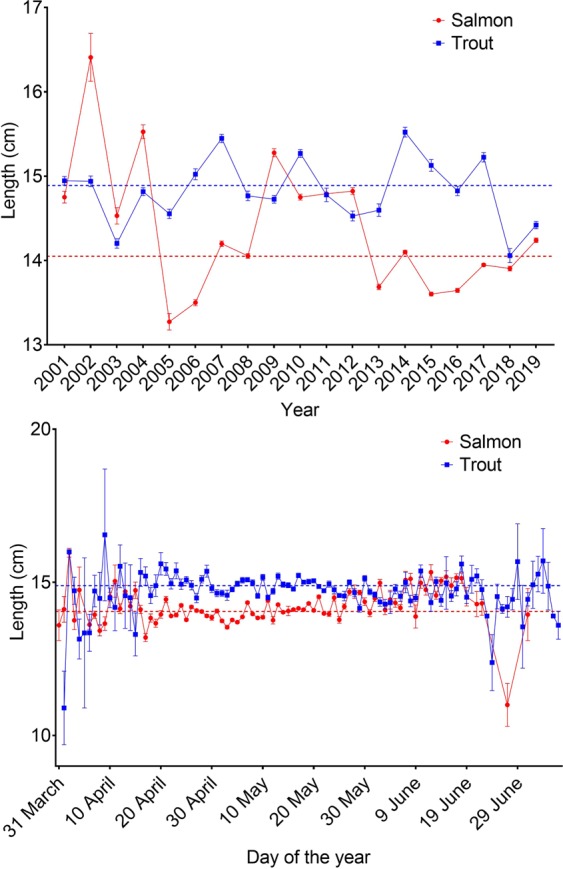
Average (± standard deviation) lengths (cm) of Atlantic salmon and sea trout migrating from the river per year and per day over the total study period (2001–2018). Dashed lines indicate average length for each species in the period.

The body lengths of each species differed across the migration periods and years (Table 4). On average, in 2005–2008, 2010, 2013–2017 and 2019 trout were larger than salmon, and salmon were larger than trout in 2002, 2004, and 2009, while in the other years there were no significant differences observed (Fig. 9). Similarly, averaged across the years, trout were larger than salmon between mid-April and mid-May (Fig. 9), while there was considerable overlap in length between the species in most years (Supplementary Fig. S10). Although the model indicates significant differences in all of the included variables, it is worth noting that the average differences in size between the trout and the salmon was not generally larger than 2 cm with considerable overlap across the migration periods and years.

**Table 4:** ANOVA output from the best fitting general linear model investigating the factors affecting length of Atlantic salmon and sea trout.

Model terms	df	Sum sq	Mean Sq	F value	P value
Day of the year	1	716	715.70	370.18	0.000
Species	1	5572	5572.00	2882.18	0.000
Year	17	3069	180.50	93.39	0.000
Day of the year x Species	1	550	550.10	284.54	0.000
Day of the year x Year	17	1192	70.10	36.26	0.000
Species x Year	17	3064	180.20	93.23	0.000
Day of the year x Species x Year	17	281	16.60	8.56	0.000
Residuals	36312	70200	1.90		

Significant variables are shown in bold, *df;* degrees of freedom, Sum.sq; Sum of squares, Mean sq; Mean sum of squares.

## Discussion

The present study used a 19-year data set, which included individual data on ~41 000 migrating salmon and trout smolts, to examine long-term smolt migration patterns and environmental cues between Atlantic salmon and sea trout. The main results can be summarised as follows: There was a shift towards an overlap in the length of the smolt migration window between the species over time, although salmon migrated out of the river an average of 8.5 days earlier than trout. The average smolt migration window was 8 weeks for salmon and 10 weeks for trout, with an average annual variation of 2 weeks for both species. The daily migrations of both species were significantly influenced by the interaction between river water temperature and water discharge. Sea water temperature outside the river mouth was also significantly associated with migration in both species. On average, body length of the species’ overlapped, but differences were observed within the migration period and among the years. Based upon these data, we conclude that salmon and trout smolts in this river have become highly synchronised and migrate in response to the same environmental cues.

In the first five years, salmon migrated in fewer numbers and over a shorter duration than sea trout (Fig. 2). This was possibly due to the salmon population in the river Guddalselva being largely derived from experimental egg planting that took place between 2003–2005 and 2008–2011, using wild, F1 hybrid and domesticated salmon from a non-native wild source and domesticated salmon^25,26^. Before these experiments, the salmon migrating out of the river most likely originated from adult salmon that had strayed from rivers in the area, and thereafter spawned in the river. On average, salmon left the river slightly earlier, and their migration numbers peaked earlier than sea trout until 2010 (Figs. 2 and 3), after which the migration timing and duration among the species became more synchronised and overlapping as the trout migration window has decreased over time. Jensen *et al*.^2^ also found an overlap in migration timing among Atlantic salmon, sea trout and Arctic char (*Salvelinus alpinus*) in their long-term study of a river in northern Norway. In addition, and similar to results from the present study, Jensen *et al*.^2^ reported that Atlantic salmon tended to migrate earlier than char and sea trout and that the range of the migration periods of salmon and trout were similar to each other. As the salmon population in the present study does not represent a native salmon population, the migration patterns of the salmon among the years do not reflect local adaptations specifically to the River Guddalselva. The first study resulting from the 2003–2005 planting experiments in the river Guddalselva found that the mean day of the year for the salmon smolt run decreased from 2005 to 2009, and that domesticated smolts left the river earlier on average than wild and hybrid smolts^25^. Similarly, the second study resulting from the 2008–2011 planting experiments found that domesticated fish migrated earliest, with wild salmon migrating on average 11.8 days later over the 2011–2015 migration periods^26^. Studies on both salmon^27^ and trout^28^ have found that domesticated individuals tend to migrate earlier than wild individuals.

Individual fish size is inherently linked to migration, as juveniles must be of an appropriate size before they can smoltify and subsequently migrate to sea^9^. Jonsson *et al*.^21^ found that larger brown trout migrated earlier than smaller conspecifics in the River Imsa, south-western Norway. Larger fish tend to be more robust to osmotic pressure, and better at evading predators than smaller fish^2^. In the present study, trout were often larger than salmon in certain times of the migration period, although this was not observed for all years (Fig. 8). Jensen *et al*.^2^ found that Atlantic salmon smolts tended to be smaller than sympatric populations of Arctic char and brown trout during their migration periods. They suggested that the difference in size between the species may reflect different selection pressures for optimal size in relation to the survival trade-off of the migration to the marine environment. For example, it may be important for salmon to reach the sea as early as possible to maximise their marine growth phase by migrating at smaller sizes or younger ages than trout or char, although this is likely a river-specific adaption. It is unclear what could have caused the fluctuating differences in average size between the species across the years. It is possible that the size of the Atlantic salmon in the present study has been influenced by the size differences between the domesticated and wild salmon smolts derived from the planting experiments detailed earlier, as the years where salmon displayed lower sizes relative to the trout coincide with the years in which the experimental fish migrated to sea. However, both studies found that the size differences were observed to be marginal between the wild and domesticated salmon^25,26,29^. In general, body length tended to overlap among the species, and size differences were not large (<2 cm) (Fig. 8).

The significant interaction between river water temperature and water discharge in the present study indicates that both these variables play an important role in migration timing in both salmonid species. In general there were fewer or no fish observed when the river temperature and discharge levels were both low or when river temperature was high, and most fish migrated out the river when river temperature was between 5–8 °C and when discharge was above 5m^3^/s (Figs. 5, S2 and S3). Therefore, in this river there appears to be an optimum range of river temperature values linked to increasing discharge values which facilitate downstream migration which is similar for both species. Freshwater temperature and discharge are both influential environmental cues for migration in both species^5,21,23,30^. River water temperature is linked to the smoltification process and metabolism and energetic requirements of growth and development^31,32^ and the influence of water temperature on smolt migration varies between rivers^4^. The river Guddalselva receives some meltwater from the Folgefonna glacier and thus often exhibits low summer water temperatures, although the optimal temperatures found in the present study were comparable to other studies^21,22,33,34^. Whalen *et al*.^15^ found that Atlantic salmon smolts in a North American river system began their migration at water temperatures of 5 °C, with peak migrations occurring at water temperatures above 8 °C. Similarly, in the river Imsa in western Norway, seaward migration of Atlantic salmon and sea trout begins at water temperatures between 5 and 11 °C^4^. Other studies have also found increasing water discharge to be a significant driver of migration^15,20,24^. Studies show that downstream migration is not totally passive and increasing water discharge can facilitate downstream movements of smolts^9,22,34^. Migration during periods of high flow may minimise the energetic costs of migration^24^. Particularly in smaller rivers, increasing water levels may facilitate movement across shallow gravel runs, and the associated increased turbidity of the water may decrease the risk of predation^34^.

Migrating salmonids need to time their entry to the sea so that the marine environmental conditions are optimal to ensure survival and maximise fitness^34^. Low sea temperatures can compromise the hypo-osmoregulatory ability of sea trout^30^. Similarly, early or late migrating smolts relative to the optimal marine conditions may face higher levels of mortality due to increased osmotic pressure or variable food conditions in the marine environment. In the present study, both salmon and trout migrations peaked when the sea water temperature just outside the river was around 10 °C (Fig. 7). Hvidsten *et al*.^13^ showed that several salmon populations along the coast of Norway left their respective rivers at different times, depending on the latitude, and that peak migration in all rivers occurred when sea water temperatures outside the rivers were above 8 °C.

In general, the optimal environmental cues for migration differ among populations and species depending on the river location, and salmonid populations may be adapted to their local environmental conditions^5,13^. Climate changes may influence sea or river water temperatures and seasonal river discharge patterns. Increasing or changing river water temperatures, changes in river discharge regimes related to changes in precipitation, snow melt or air temperature are potential effects of climate change at this latitude^35^. Such changes may affect the survival of migrating smolts, and lead to a shift in migration timing and duration by altering important life history traits by influencing metabolic or reproductive processes^36^, like smoltification or egg development, or indirectly through resource availability^37^. The present study observed variation in the mean migration day of both salmon and trout smolts across the years, although there was no observable long-term trend apart from a decrease in the length of the trout migration window resulting in a synchronisation of the migration period with the salmon. Increasing river water temperatures or increasing water discharge due to a warmer climate may cause the smolt migration window in the present river system to shift to earlier or later in the season, with unknown consequences on smolt survival and recruitment.

Models predict changes in several aspects of fish life history as a result of climate change, and several long-term studies have observed a shift in the distribution of marine stocks^35^ and in the timing of key life history traits for salmonids^17,18,38^. While Byrne *et al*.^20^ found that the timing of smolt migration over a 30-year period in a river in western Ireland was consistent over time, a study by Dempson *et al*.^39^ found that migration timing of Atlantic salmon in several Canadian rivers had changed in relation to warmer climate conditions over a 35 year period. Similarly, Otero *et al*.^38^ found that the migration timing of Atlantic salmon across their North Atlantic range has changed in response to changing global climate in the last five decades.

The effects of climate change on fish populations may cause conflicting selection pressures in different life stages^19^, and may affect locally adapted populations in different ways. Therefore, it is difficult to predict an overall effect of climate change due to this complexity. Along with sustained changes in climate and their expected influence on migration patterns, extreme climate events, such as flooding or drought conditions can also be expected to influence migration through decreased survival and recruitment^30^. Long term studies of salmon and trout migration are few, with some showing a consistency in the influence of environmental variables on migration timing^20,21^. Long term monitoring of environmental factors that influence salmonid migration patterns are important for understanding local population adaptions, and for predicting the likely effects of climate change on salmonid populations.

## Methods

### Study area

The river Guddalselva is located in the middle region of the Hardangerfjord in western Norway (59 °96′N, 5 °99E) (Fig. 1). The river drains an area of 37 km^2^, with a downstream section of approximately 2 km available for anadromous species, after which there is an impassable waterfall (Liarefossen). The river supports a small population of sea trout below the waterfall, and annual catch reports for Atlantic salmon from 1968 to 2008 indicate that Atlantic salmon are documented periodically, although no self-sustaining population exists^25,40^. In 2000, an experimental field station of the Institute of Marine Research (IMR) was established to study the interaction between aquaculture and wild salmonids in collaboration with river owners and management authorities^41^. A Wolf trap was installed on the river in the winter of 2000, allowing precise data on the downstream migration of salmonids. The trap is assembled each year in March before the beginning of the smolt run and dismantled once the smolt run has terminated later in the year.

From 2003 to 2005, and 2008 to 2011, in collaboration with local river owners and management authorities, the IMR planted out ~half a million eyed eggs from a total of 144 families of wild, F1 hybrid (domestic-wild cross) and domesticated Atlantic salmon. These eggs were planted to study survival and fitness-related trait differences among the families in a natural environment. For details see^25^ for the 2003–2005 study and^40^ for the 2008–2011 study. The wild populations used in the two studies were translocations from the river Lærdal (2003–2005 study) and the river Etne (2008–2011 study). Therefore, the migrating salmon smolts investigated in the present study represent individuals derived from these long-term egg planting experiments, in addition to naturally recruited salmon smolts that are the offspring of wild salmon straying from neighbouring populations in the region. Thus, the salmon smolts investigated here cannot be considered as “locally adapted” to the river Guddalselva.

### The data

The data consisted of daily counts of Atlantic salmon and sea trout that descended the river from the period April to June from 2001 to 2019. Biological data (weight, length, condition factor) were recorded for each fish. Environmental data pertaining to the river temperature (°C) and river water discharge (m^3^/s) for each day during the same period were obtained from NVE.NO (the Norwegian Water Resources and Energy Directorate). Daily modelled average sea water temperatures (°C) were estimated from monthly temperature loggers in the area near the river Guddalselva. Environmental data were missing for certain years during the study period: 2002–2006 and 2008 for river water temperature, 2002 and 2006 for river water discharge. Therefore, it was decided to run several analyses for different time periods depending on the availability of environmental data. The interaction between river temperature and discharge was investigated for the period 2009–2019, while the influences of discharge and sea water temperature were investigated separately for the period 2001, 2003–2005 and 2007–2019. The two water temperature variables were highly correlated over all the years (r = 0.91, df = 1056, p = < 2.2e-16). Prior to statistical analysis, individuals with missing data or extreme biological values resulting from likely recording errors were removed from the dataset. Body length was the most frequent biological measurement and was therefore used to examine any size differences between the species throughout the entire study period.

### Daily numbers of salmonids leaving the river

Mixed-effects zero-inflated regression models were used to investigate the potential drivers of outward migration of salmon and sea trout separately using the *glmmTMB* function from the glmmTMB package in R^42,43^. A glmmTMB model is comprised of 3 main components: a conditional model formula and its distribution relating to the fixed and random effects, a dispersion model formula relating to the dispersion parameter of the conditional model distribution, and a zero-inflation model which relates to the probability of observing a zero that is not caused by the conditional model^43^. Due to logistical constraints, the smolt migration trap was not checked daily and therefore the daily numbers of salmon and trout were estimated using a smoothing regression for each day in the period April – June within each year. The response variables were the estimated daily count of salmon and sea trout each year respectively, and both were modelled using a negative binomial distribution with a log-link function. The models included a dispersion parameter that varied over the years to account for increasing variation in the response over time. Zero-inflated models were chosen to control the over-dispersion in the data arising from the large number of zeros, while also allowing for the investigation of the potential processes underlying the observed zero counts.

In the full model investigating river temperature and river discharge, the fixed part of the model contained daily water temperature, daily water discharge and year and all and two-way interactions between these three variables (1.1). The former two variables were modelled as continuous explanatory variables and year was modelled as a factor explanatory variable. The year and the change in daily water discharge (flow decreasing; negative, or flow increasing; positive), and their interaction were included as factor explanatory variables. In the full model investigating river discharge and sea water temperature, the fixed part of the model contained discharge as a continuous explanatory variable interacting with year as a factor variable, sea water temperature as a continuous variable interacting with year, and year and change in daily discharge as above (1.2).

Both river and sea water temperature were highly correlated with day of the year (river: r = 0.90, df = 1056, p = < 2.2e-16; sea: r = 0.89, df = 1056, p = < 2.2e-16), therefore day of the year was not included as a variable in these analyses. Week number nested within year was included in each model as a random effect to allow for correlation within weeks across the years. The zero-inflation component of model 1.1 contained the continuous variables of daily water temperature and discharge, while the zero-inflation component of model 1.2 contained daily discharge only. For both salmon and trout, the full models were as follows:

Count ~ Year x Change in Discharge + River Temperature x Year + Discharge x Year + Discharge x River Temperature + (1| Year/Week) + disp(Year) + (ZI ~ River Temperature + Discharge) (1.1)

Count ~ Year x Change in Discharge + Discharge x Year + Sea Temperature x Year + (1| Year/Week) + disp(Year) + (ZI ~ Discharge) (1.2)

The fit of the above models was assessed by plots of the Pearson residuals against fitted values and model covariates. The *Anova* function from the car package^44^ was used for each of the above models to assess the significance of the fixed model terms.

### Length of salmonids leaving the river

To investigate whether body length differed among the species across the years and within the migration period, a general linear model was implemented using the *lm* function in R. The response variable was the length of each fish, modelled using a Gaussian distribution. Day of the year was included in the model as a continuous explanatory variable in a three-way interaction with the factor variables of year (2001–2019) and species (salmon or trout).

Length ~ Day of the Year x Species x Year (1.2)

Model fit was assessed by examining a histogram of the residuals and plots of residuals against fitted values and model covariates. The *anova* function was used to assess the significance of the model terms as above.

### Ethical statement

Experimental protocol (permit numbers S.nr. 07/13020-1; S.nr. 2015/33655; S.nr. 17/15858) were approved by the Norwegian Animal Research Authority (NARA). All welfare and use of animals were performed in strict accordance with the Norwegian Animal Welfare Act of 19^th^ June 2009, enforced on the 1^st^ of January 2010. In addition, all personnel involved in the data collection had undergone training approved by the Norwegian Food Safety Authority, which is mandatory for all personnel handling fish.

## Supplementary information

Supplementary File 1.

Supplementary File 2.

## Data Availability

All data generated or analysed during this study are included in this published article as Supplementary File 2.
