# Supplementary material for: Atlantic salmon and sea trout display synchronised smolt migration relative to linked environmental cues
Source: Sci Rep. 2020 Feb 26;10:3529. doi: 10.1038/s41598-020-60588-0 (PMC7044379; doi:10.1038/s41598-020-60588-0)
Supplement: Supplementary file 1 [file 41598_2020_60588_MOESM1_ESM.docx]

**Atlantic salmon and sea trout display synchronised smolt migration relative to linked environmental cues**

Alison C. Harvey^1*^, Kevin A. Glover^1, 2^, Vidar Wennevik^1^ & Øystein Skaala^1^

^1^ Institute of Marine Research (IMR), Bergen, Norway

[alison.harvey@hi.no](mailto:alison.harvey@hi.no), [kevin.glover@hi.no](mailto:kevin.glover@hi.no), vidar.wennevik@hi.no, [oystein.skaala@hi.no](mailto:oystein.skaala@hi.no),

^2^ Institute of Biology, University of Bergen, Norway

* Corresponding author

**Supplementary Tables & Figures**


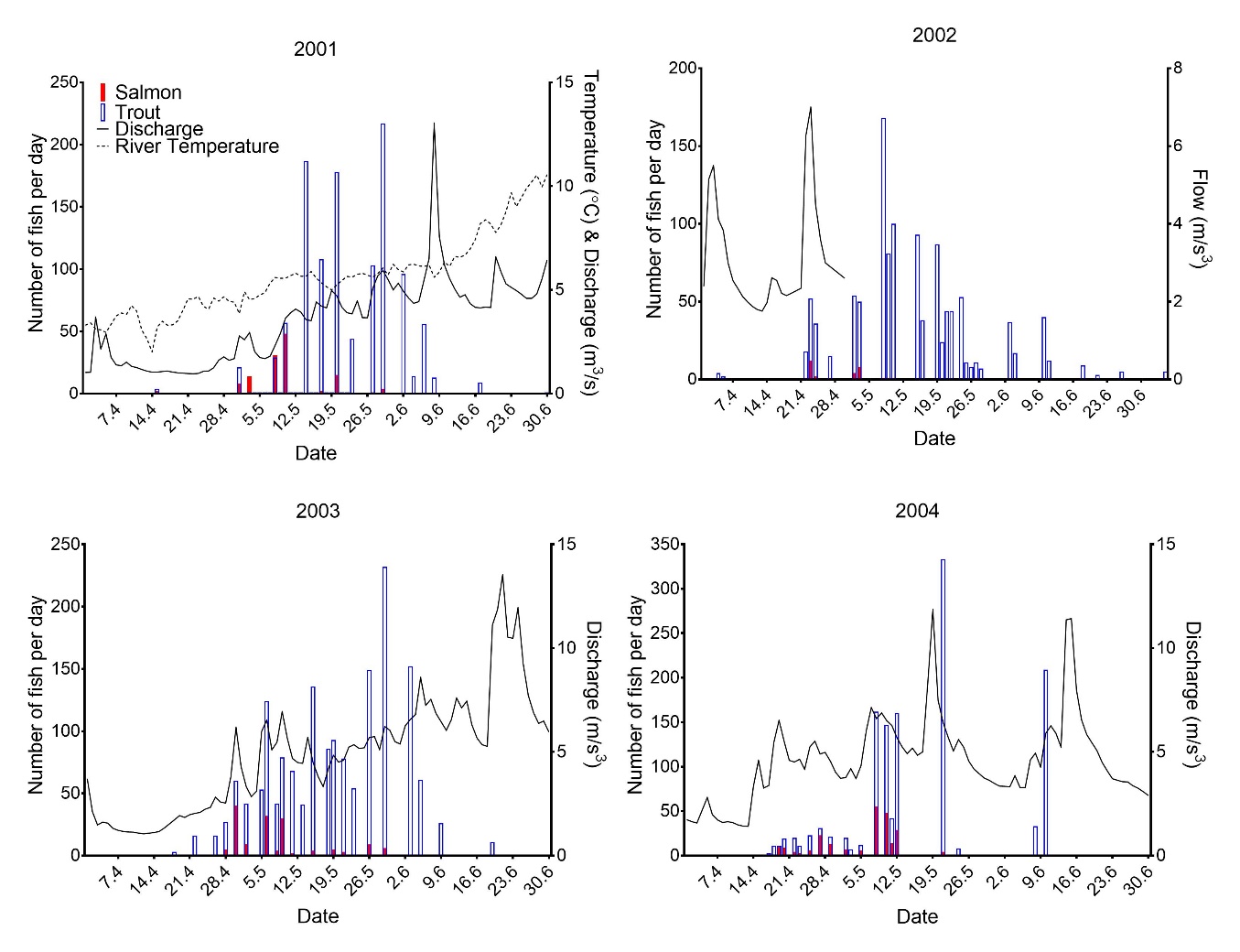

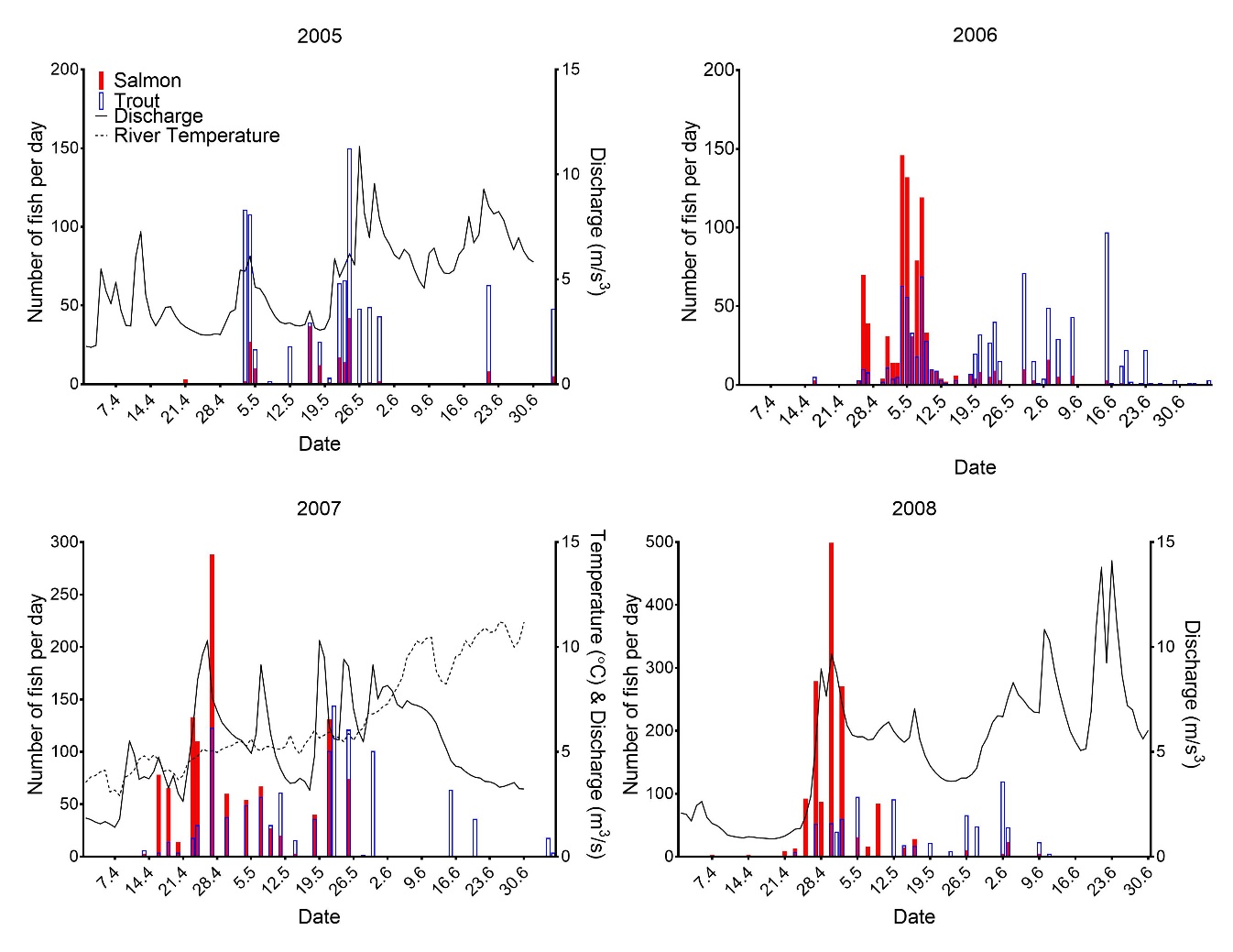


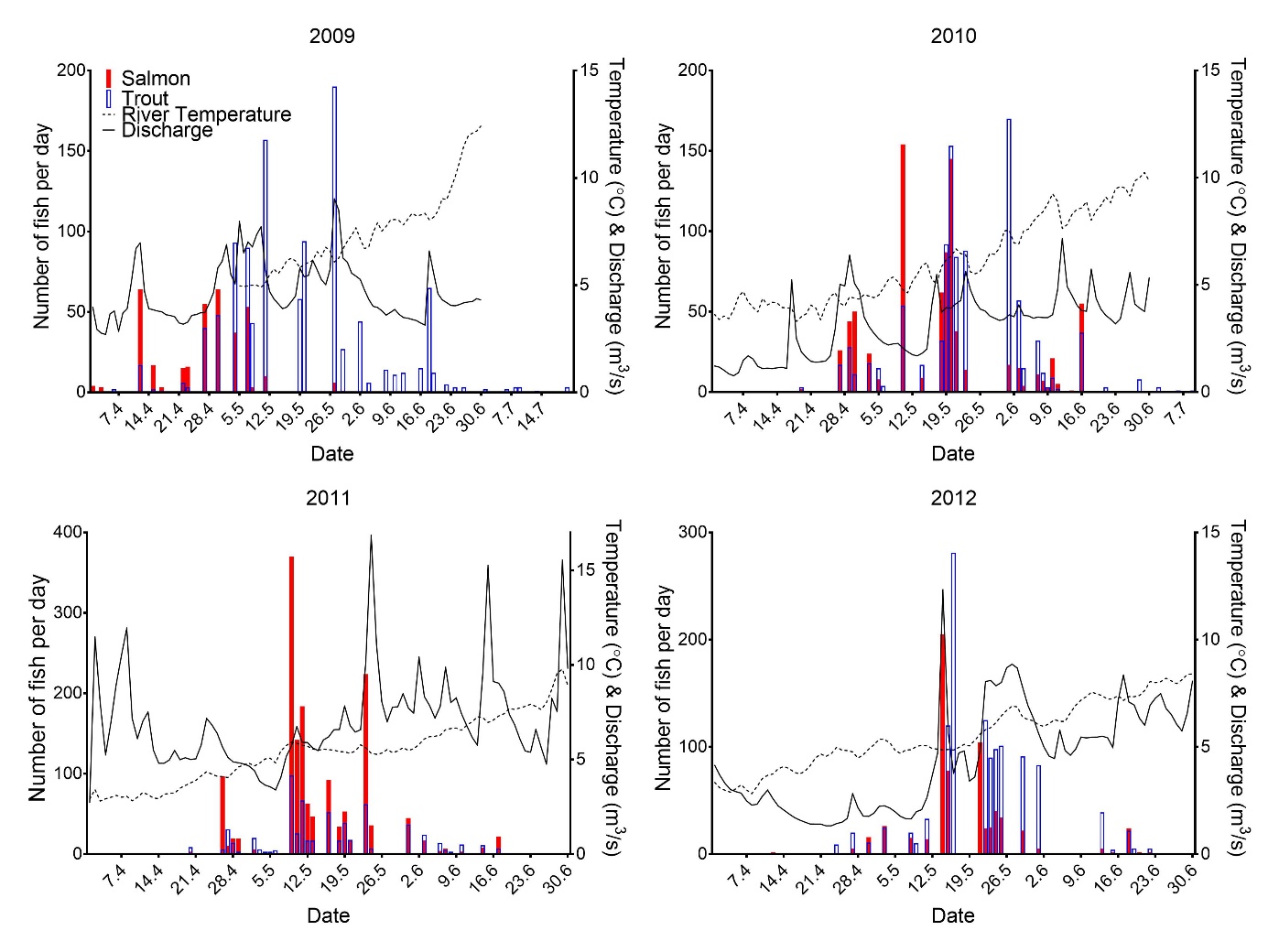

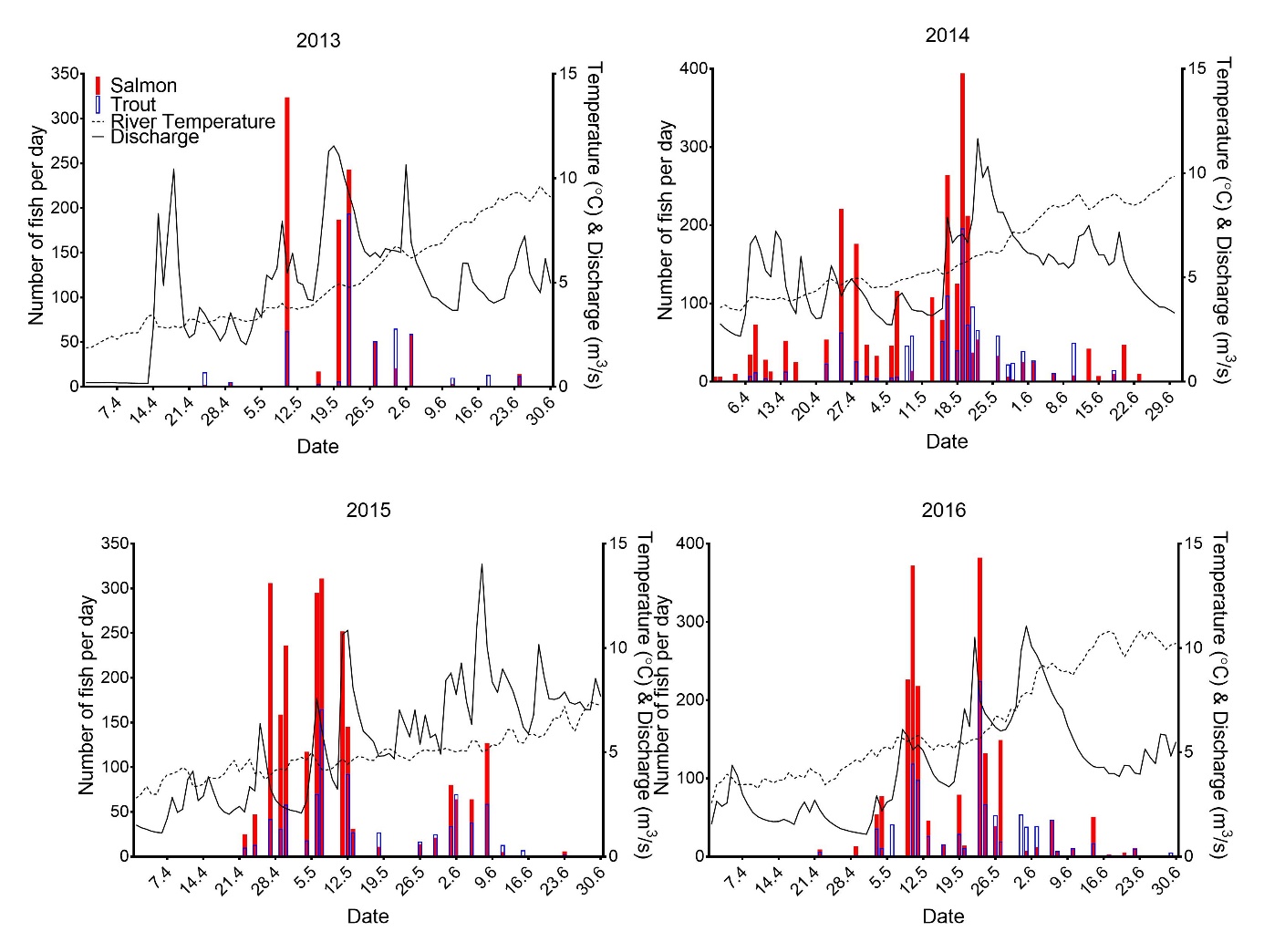

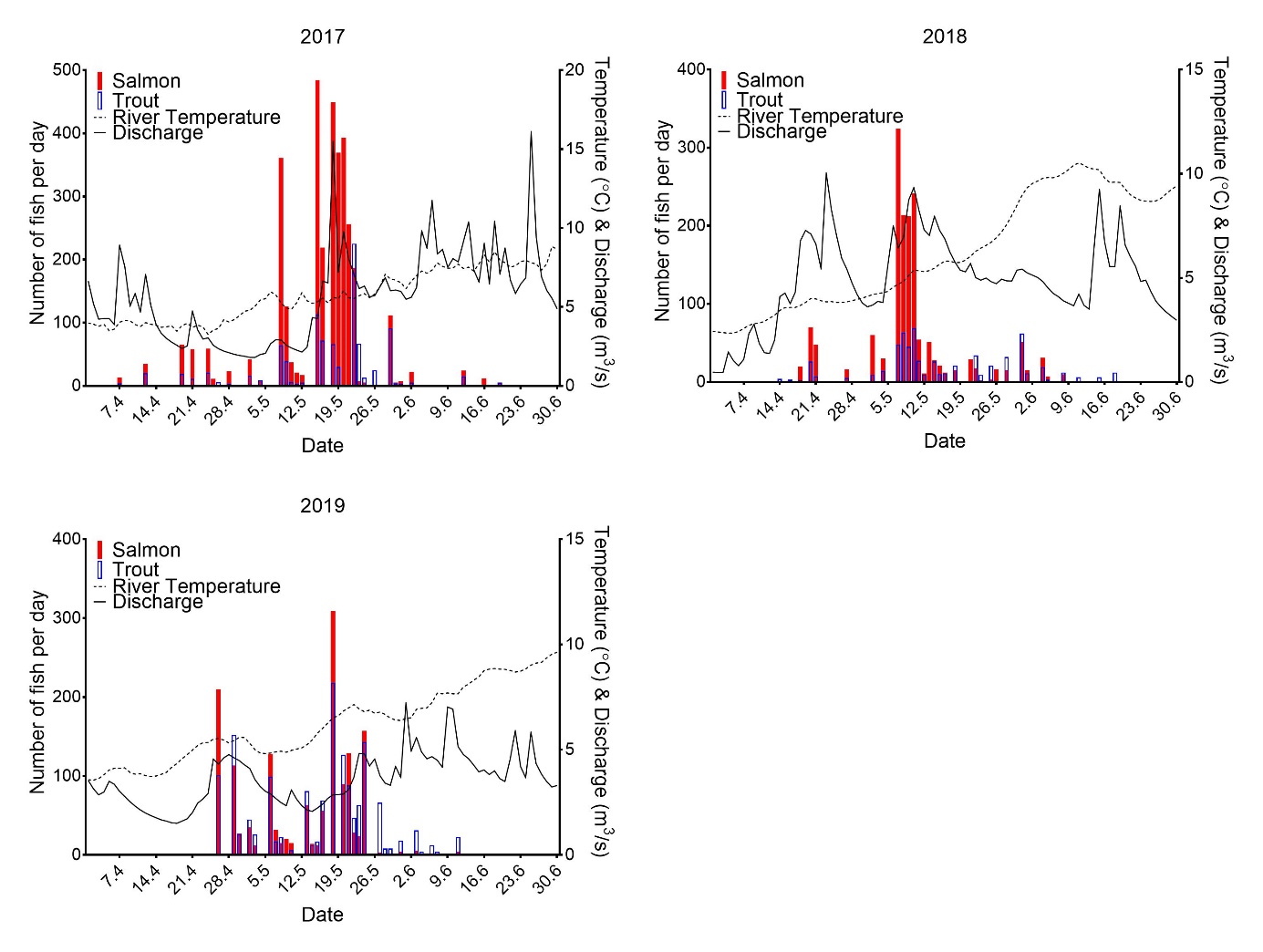
Figure S1: Daily numbers of Atlantic salmon and sea trout migrating from the river over the total study period (2001 – 2019). Daily river water temperature (°C) and daily water discharge (m/s^3^) are also shown.


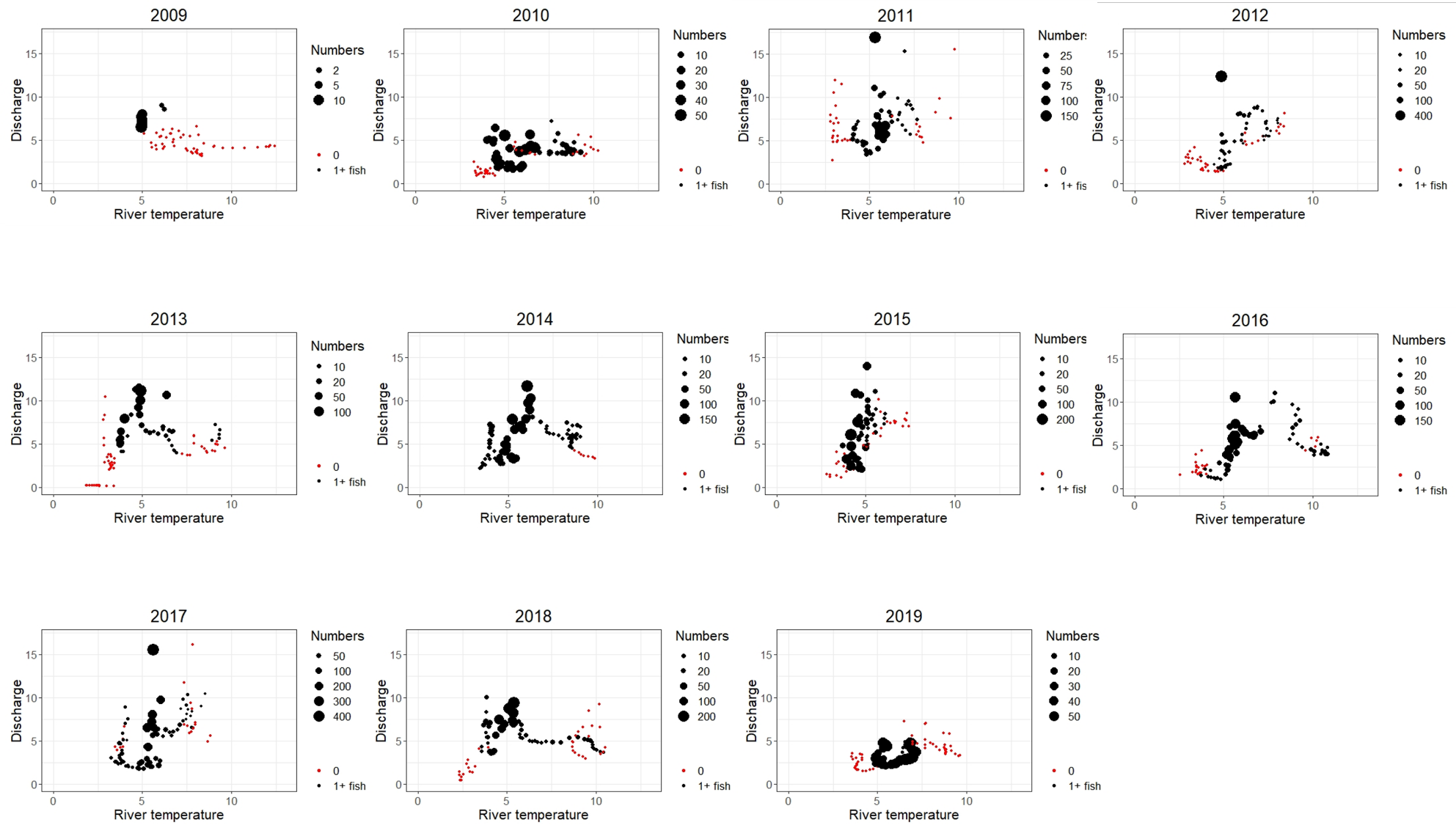


Figure S2: Number of salmon migrating out of the river per year for the period 2009 – 2019 in relation to river water temperature (°C) and water discharge (m^3^/s). Red dots indicate days where no salmon were migrating, black dots indicate days where one or more salmon was migrating out the river. The size of the dots is an indication of the number of salmon migrating that day, where larger dots indicate higher numbers of salmon.


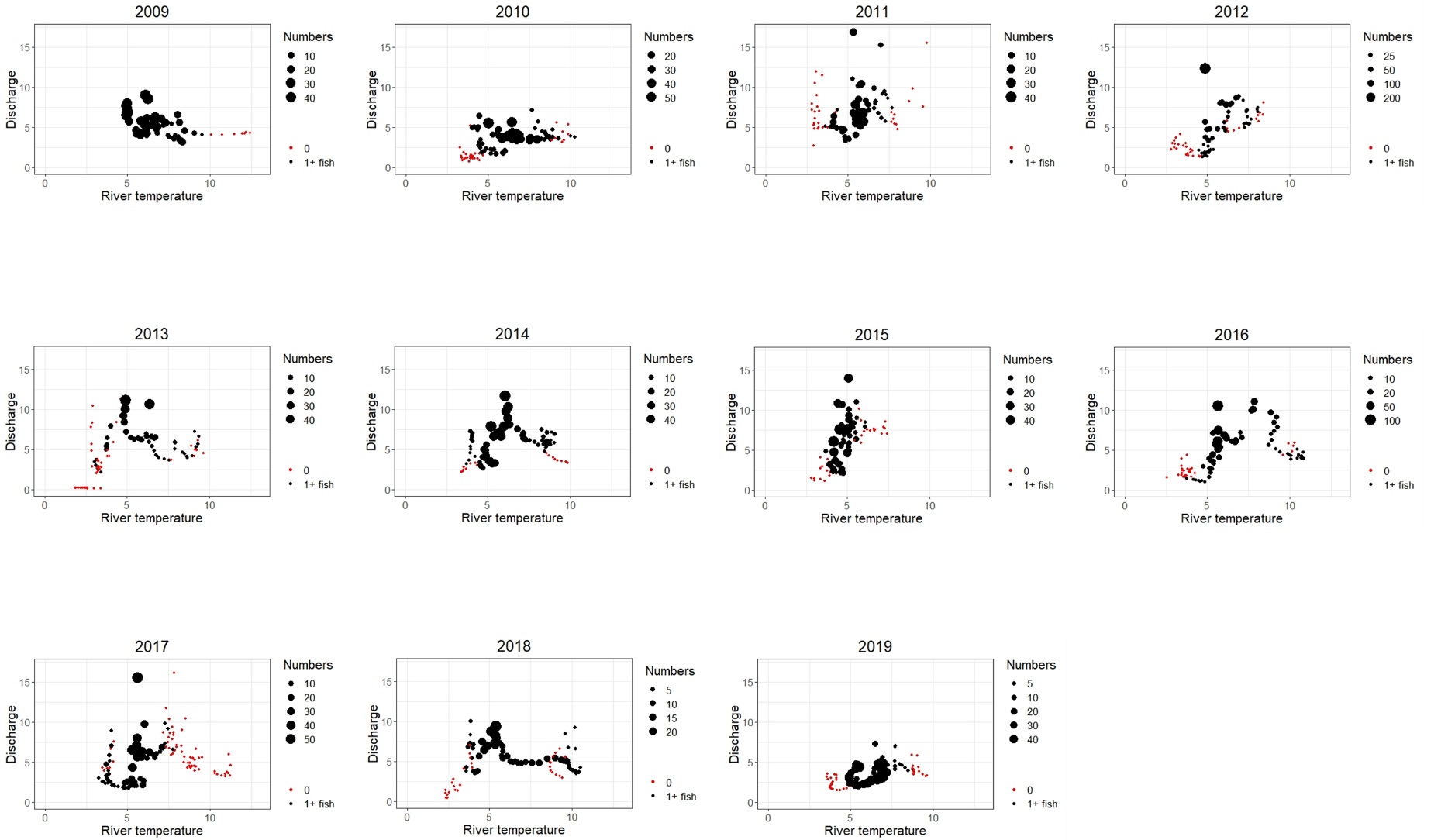


Figure S3: Number of trout migrating out of the river per year for the period 2009 – 2019 in relation to river water temperature (°C) and water discharge (m^3^/s). Red dots indicate days where no trout were observed, black dots indicate days where one or more trout was migrating out the river. The size of the dots is an indication of the number of trout migrating that day, where larger dots indicate higher numbers of trout.


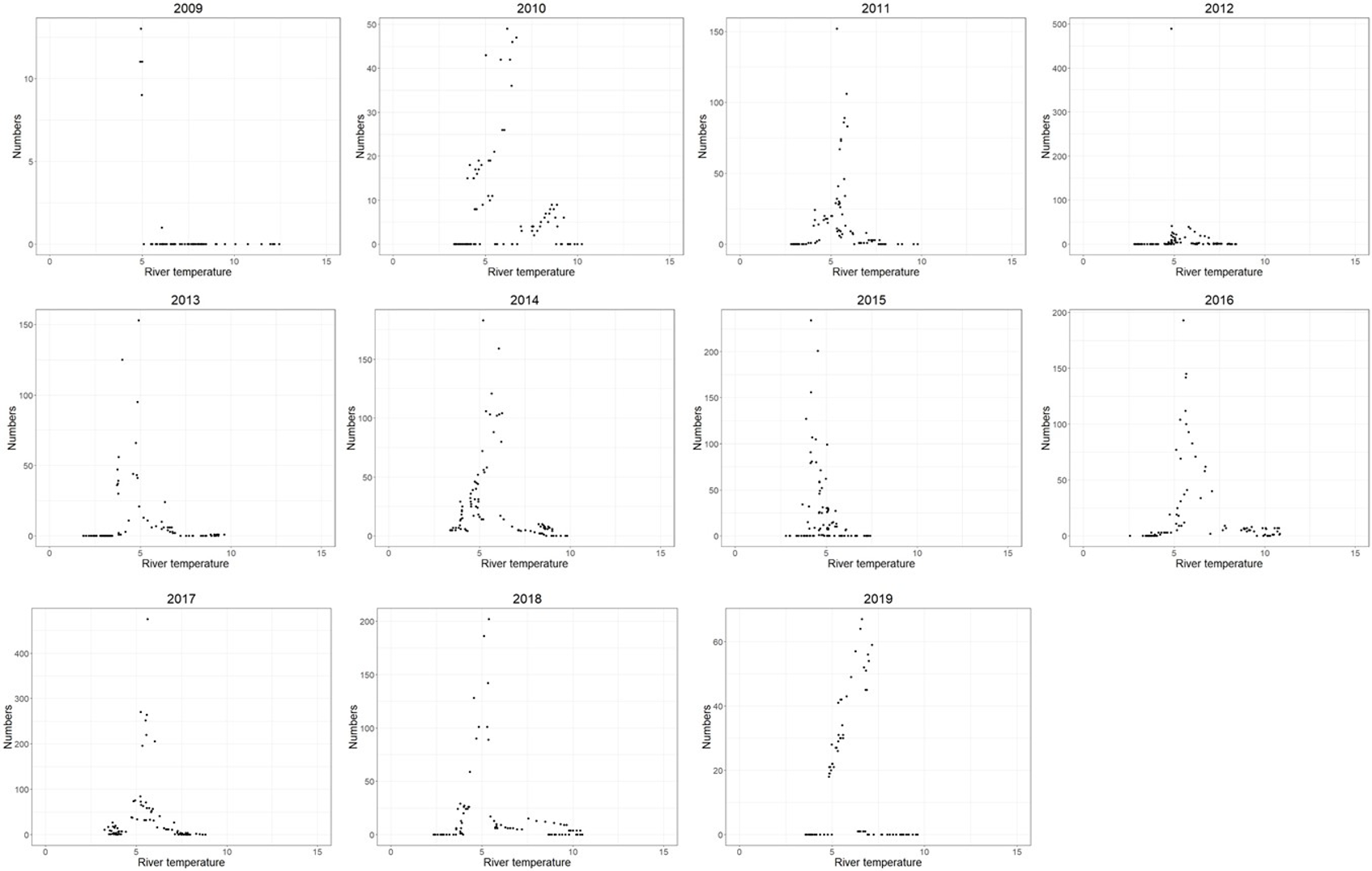


Figure S4: The number of salmon migrating out of the river per year in relation to river water temperature (°C).


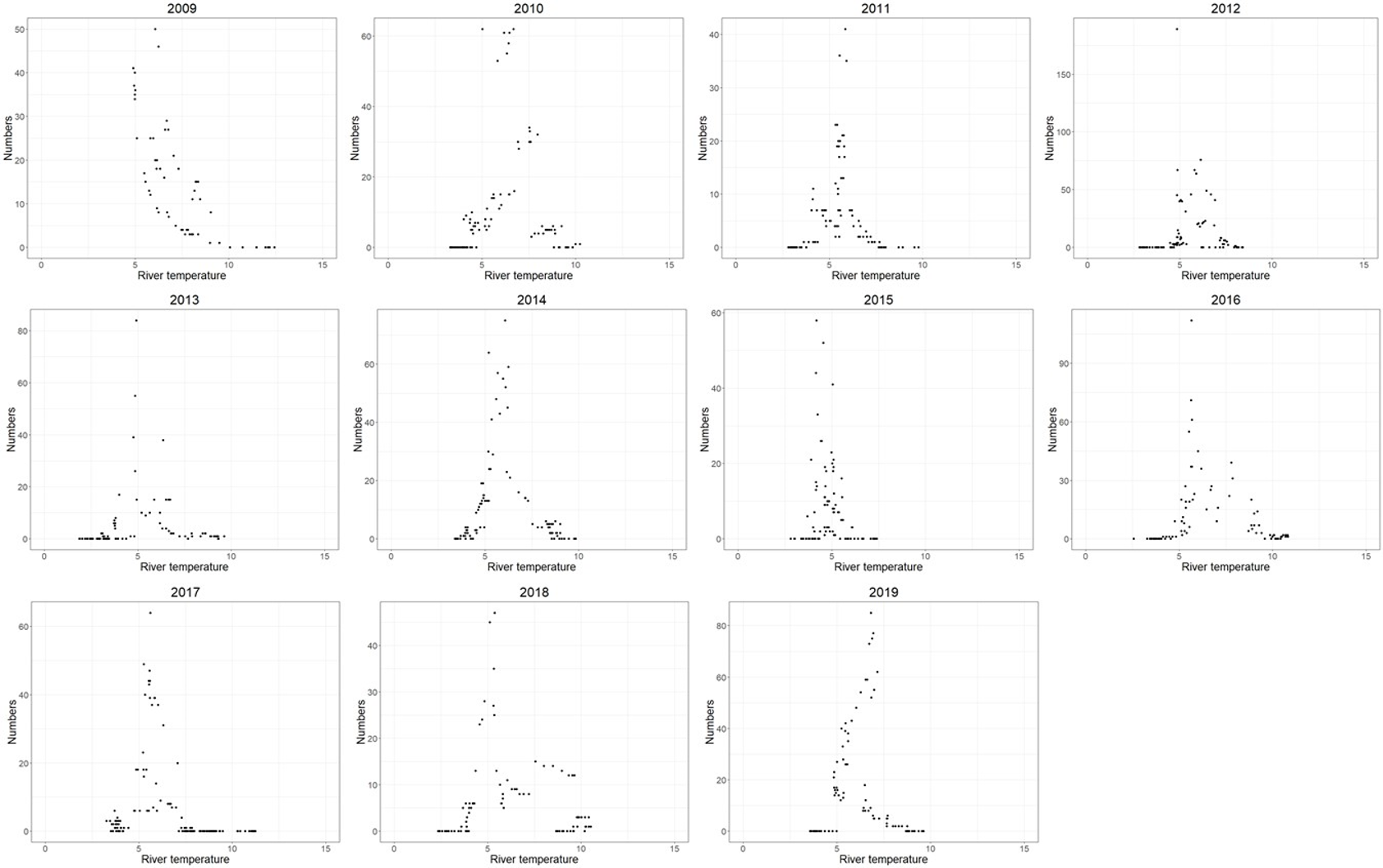


Figure S5: The number of trout migrating out of the river per year in relation to river water temperature (°C).


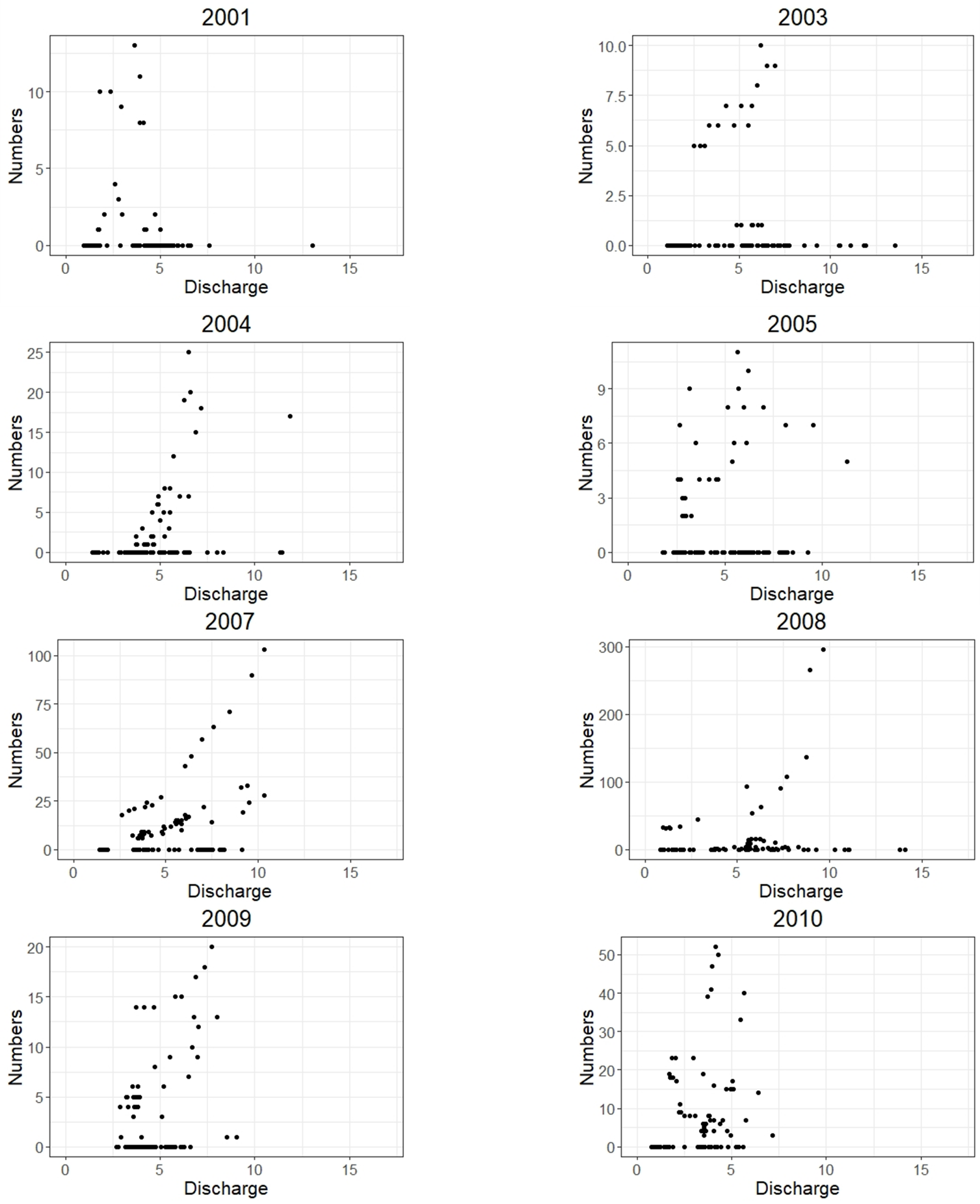


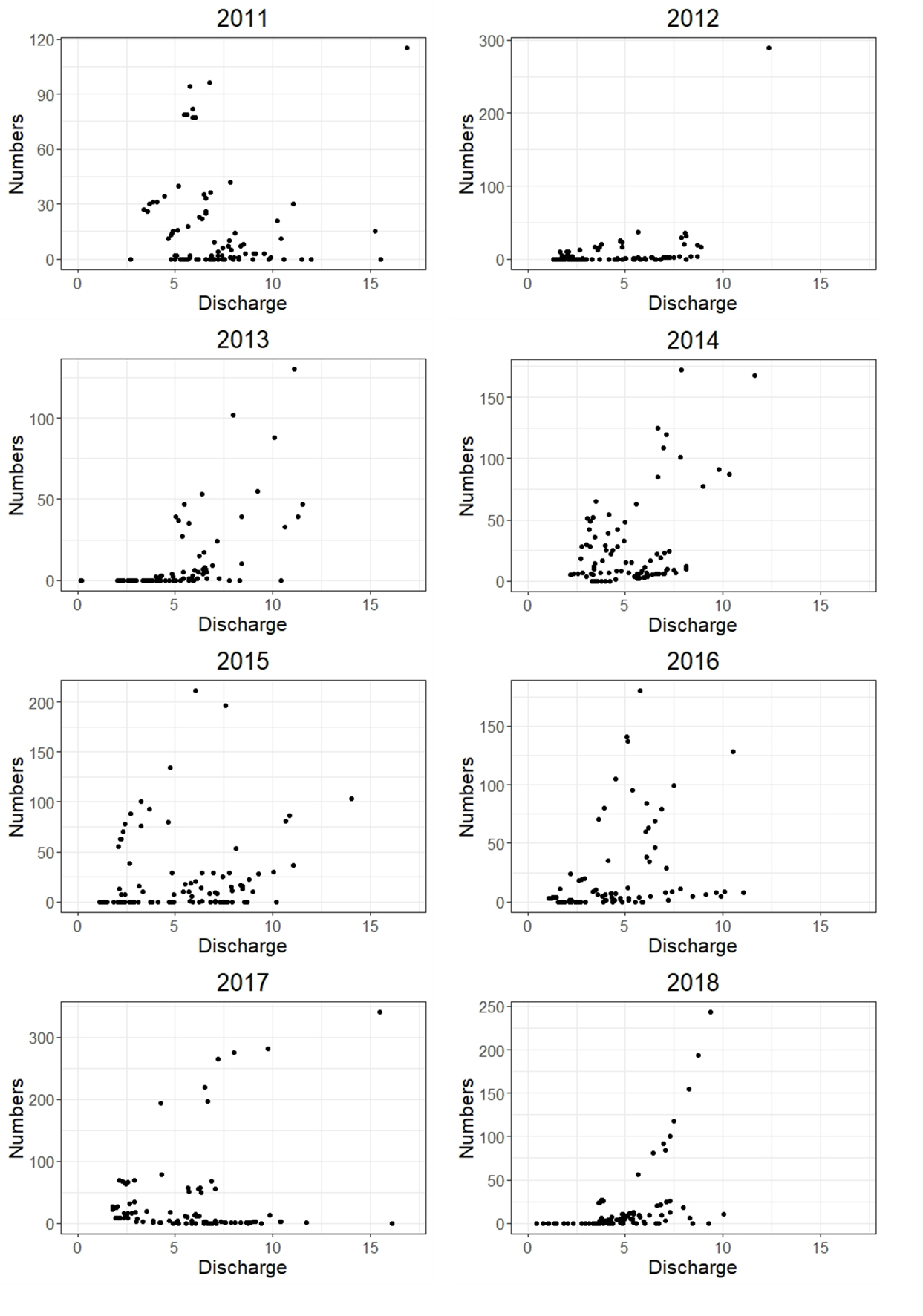


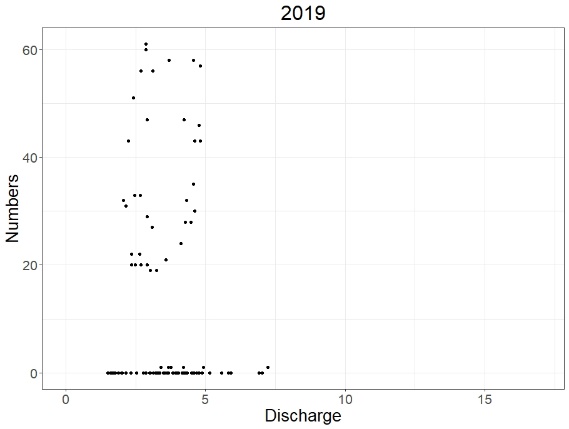


Figure S6: The number of salmon migrating out of the river per year in relation to water discharge (m^3^/s).


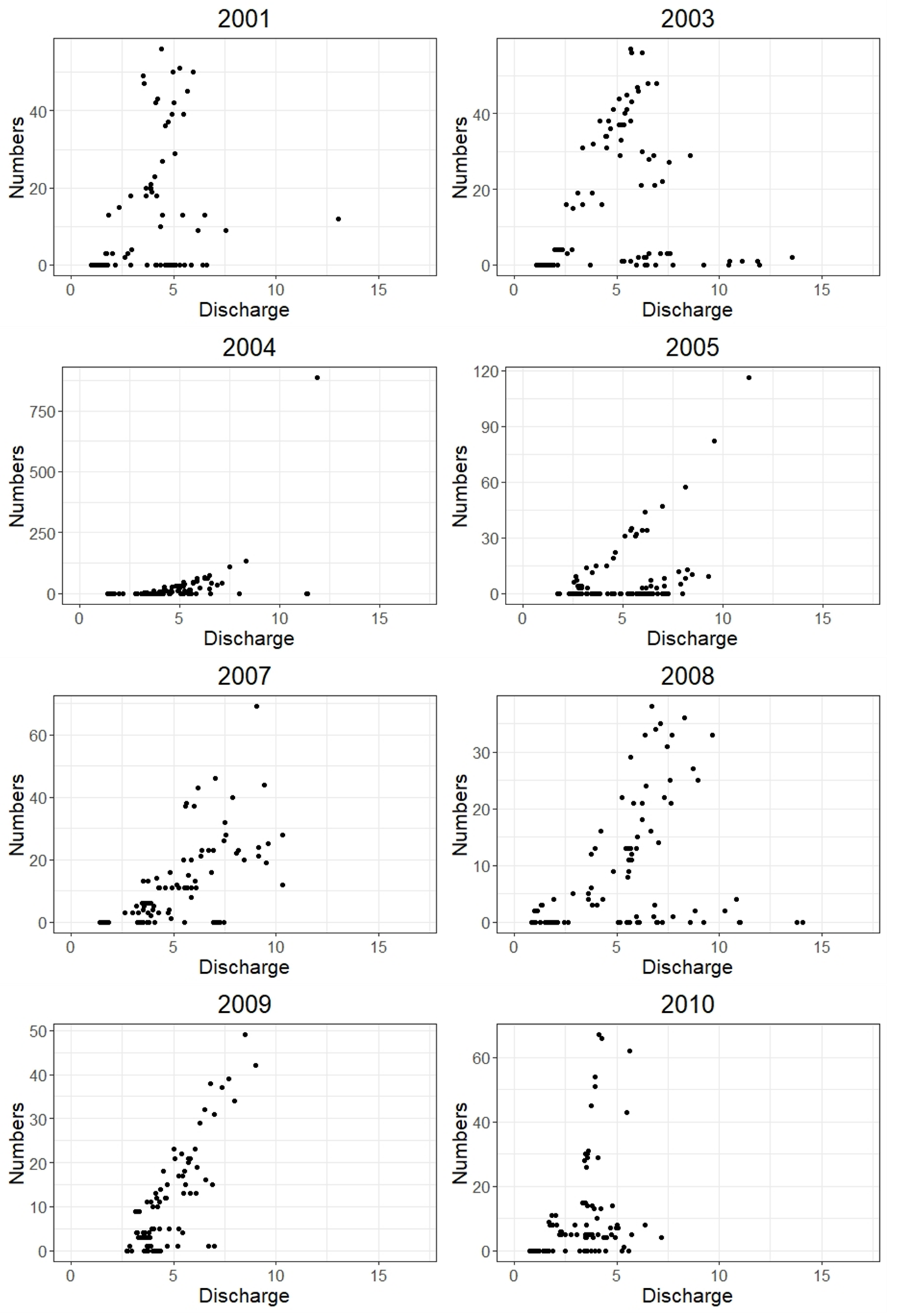


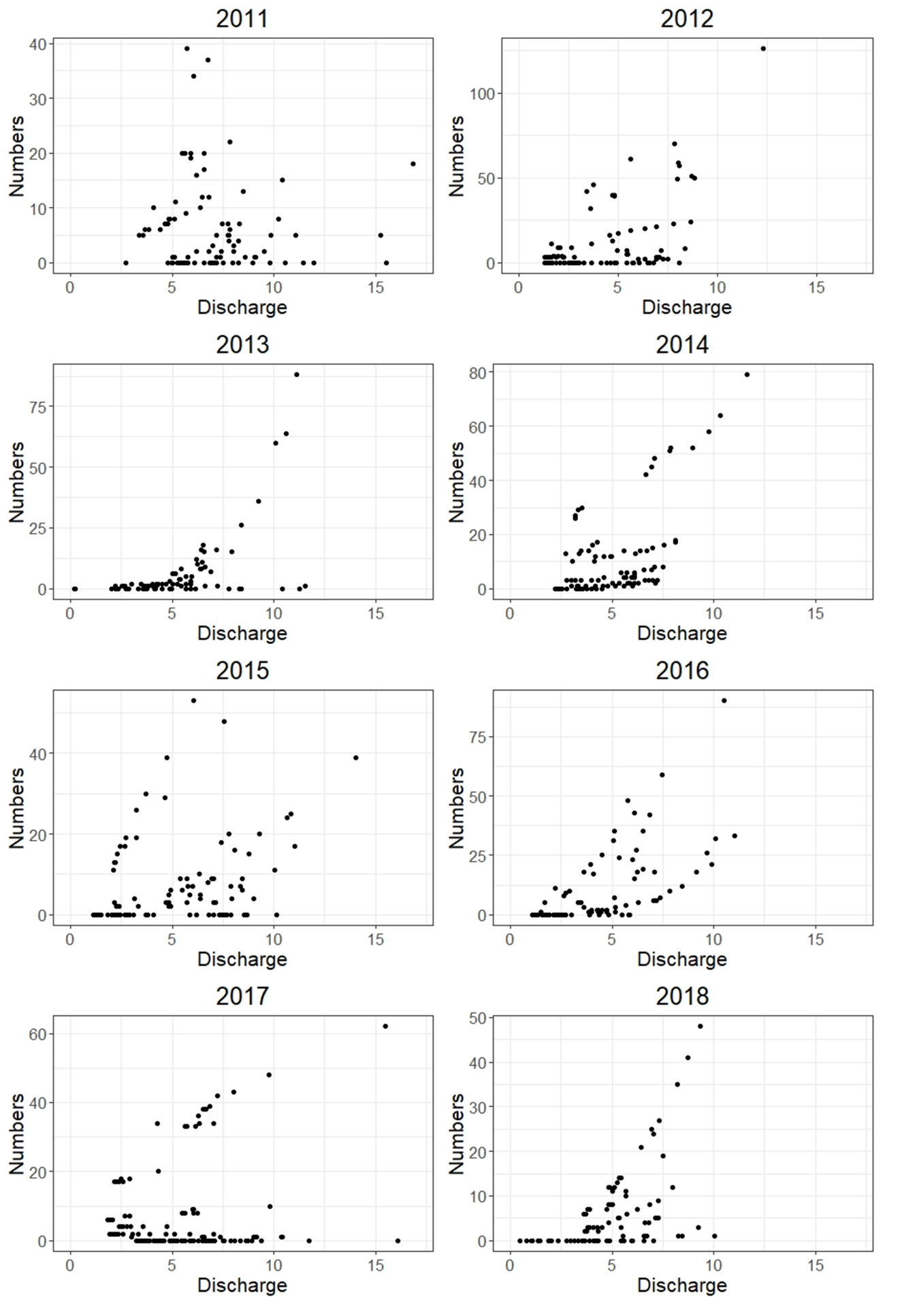


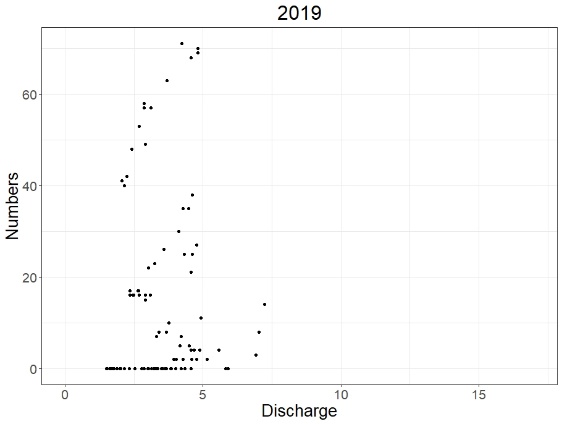


Figure S7: The number of trout migrating out of the river per year in relation to water discharge (m^3^/s).


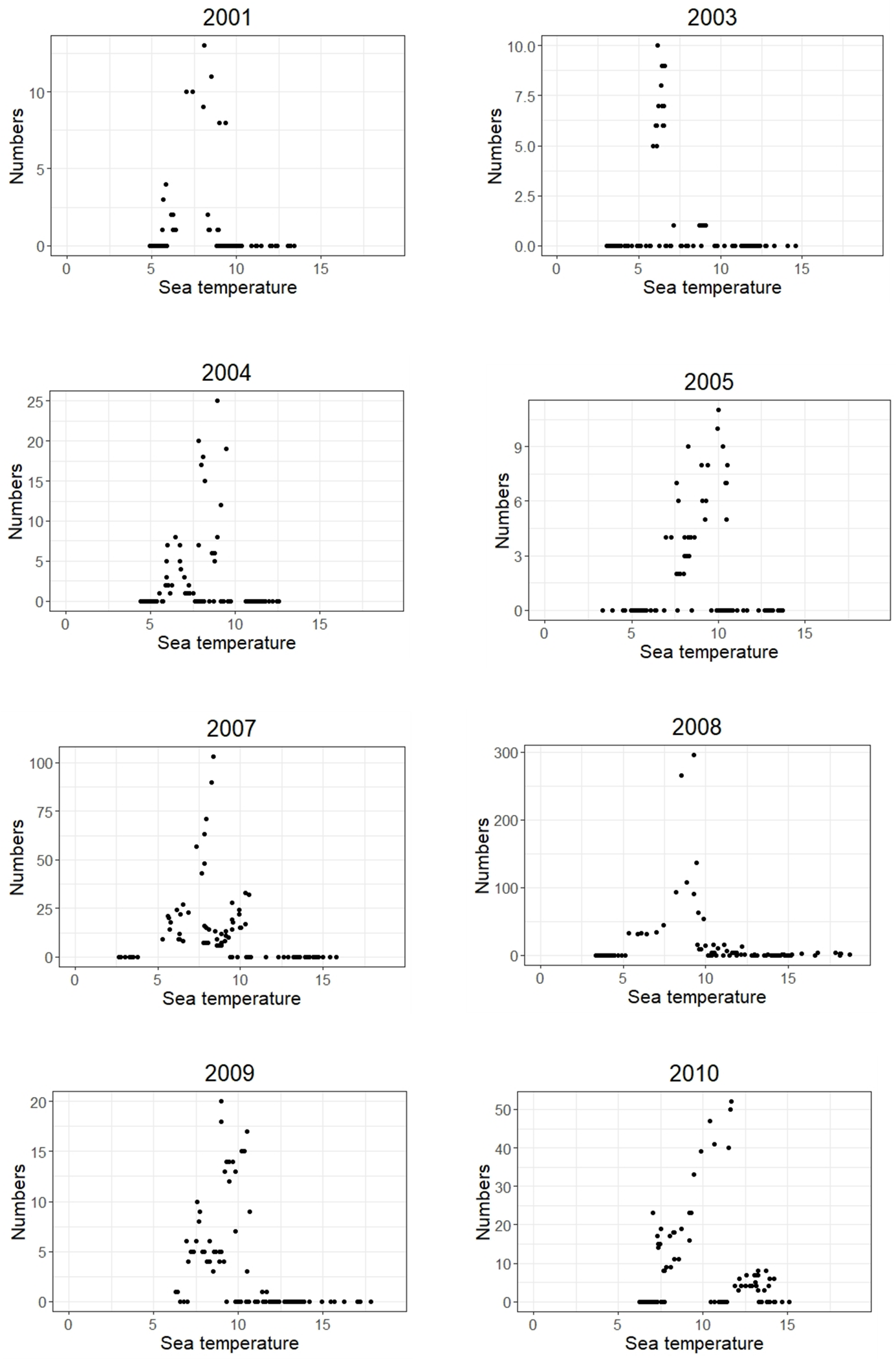


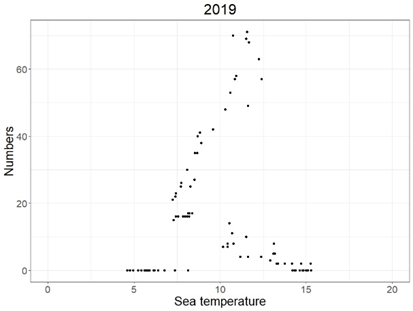

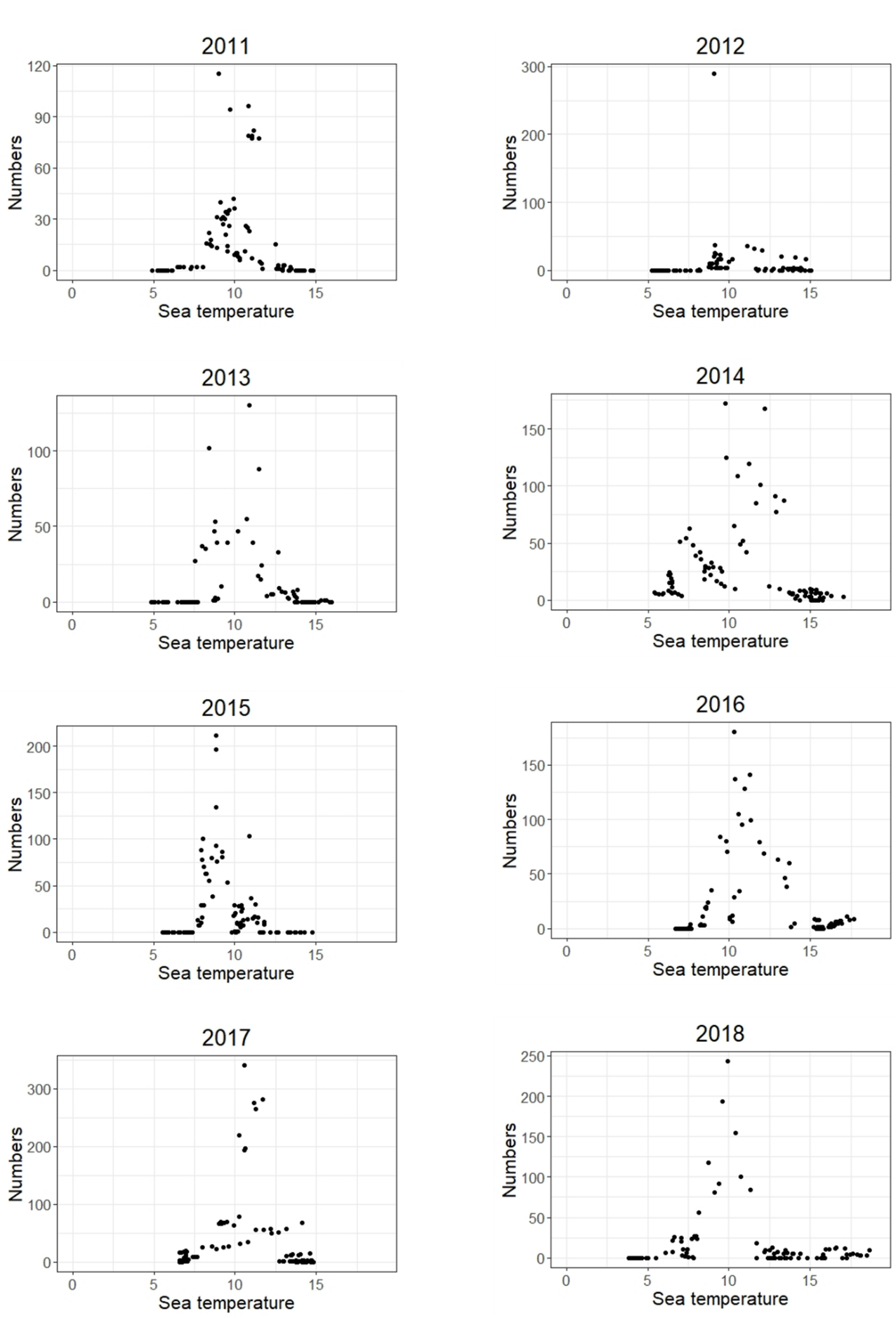


Figure S8: The number of salmon migrating out of the river per year in relation to sea water temperature (°C).


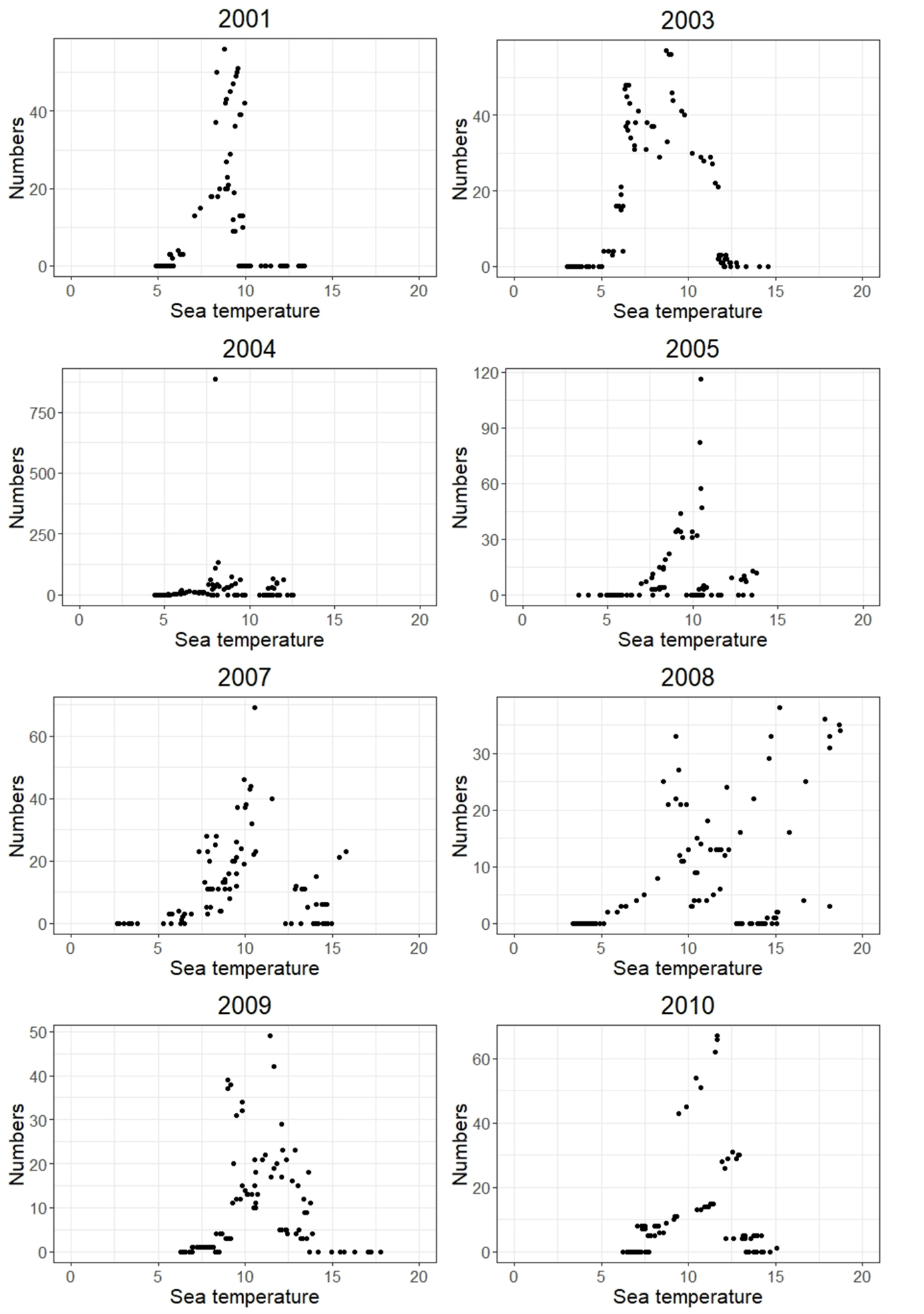


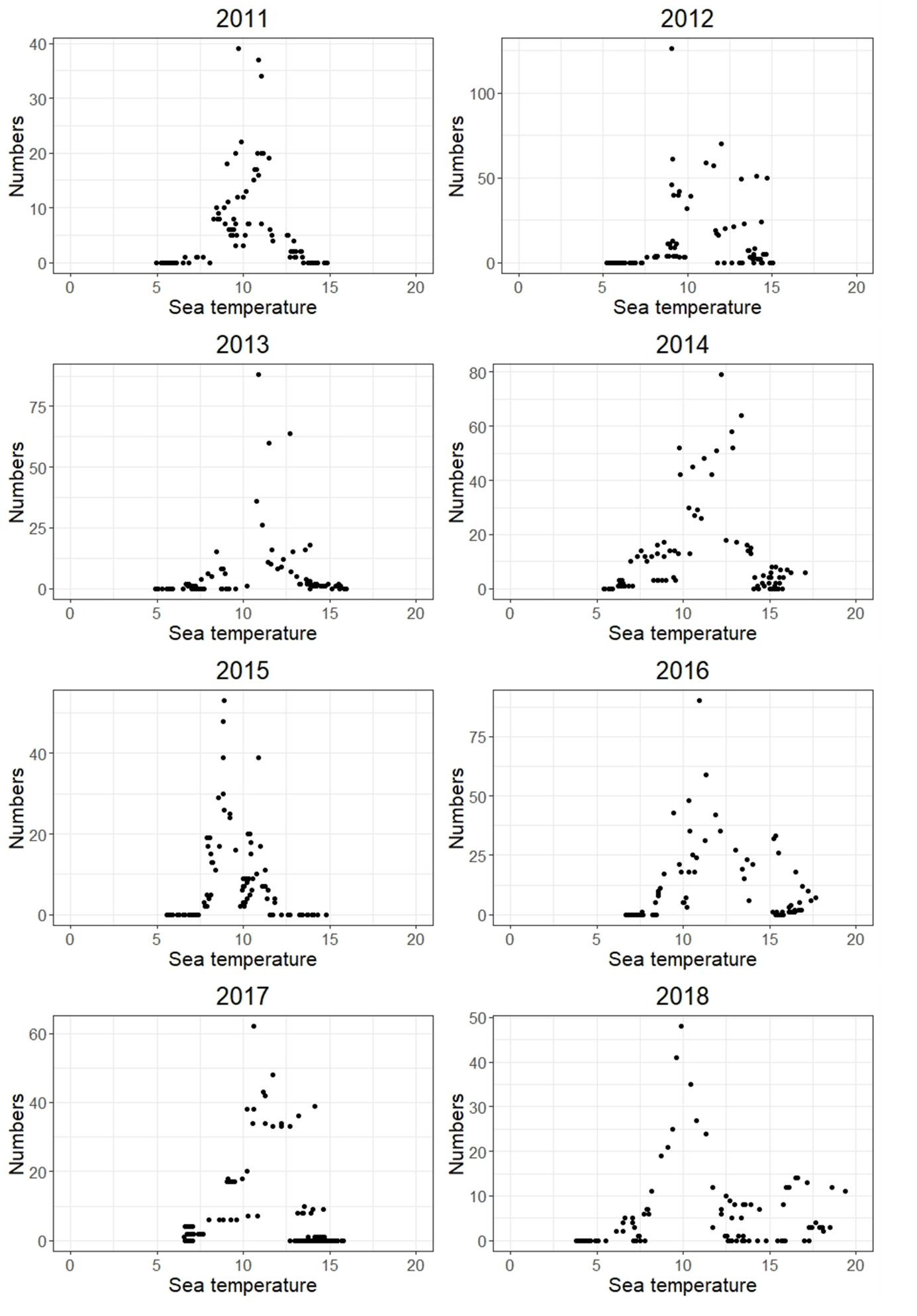


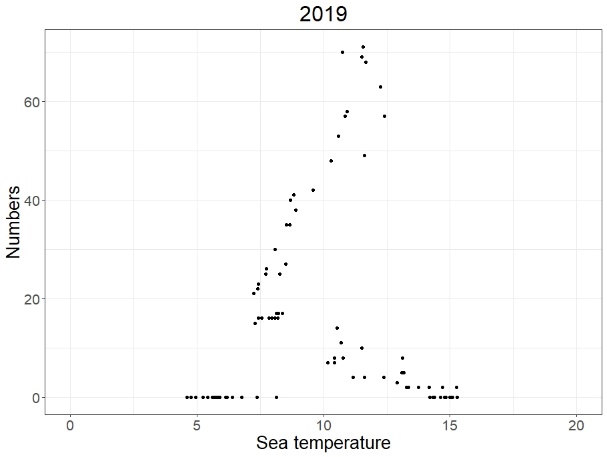


Figure S9: The number of trout migrating out of the river per year in relation to sea water temperature (°C).


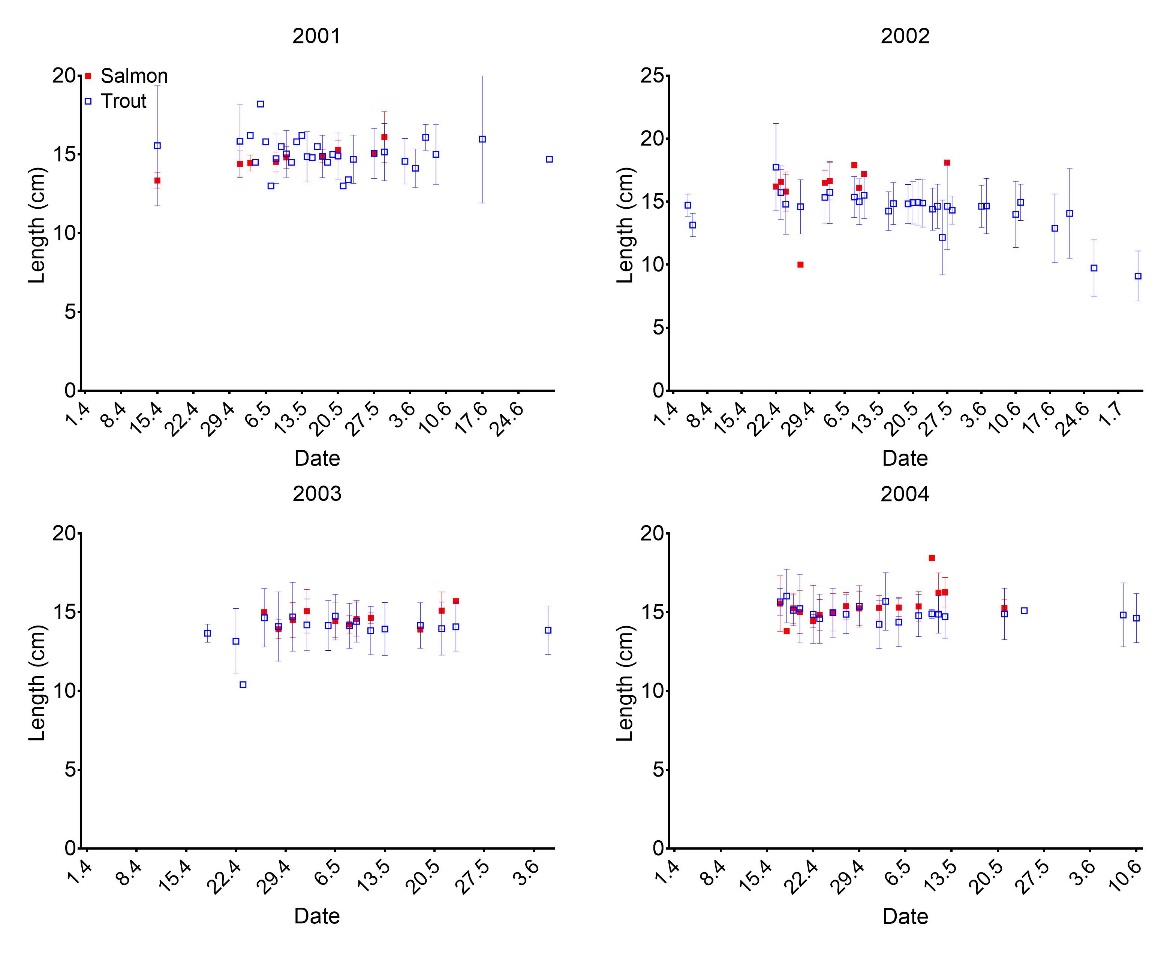


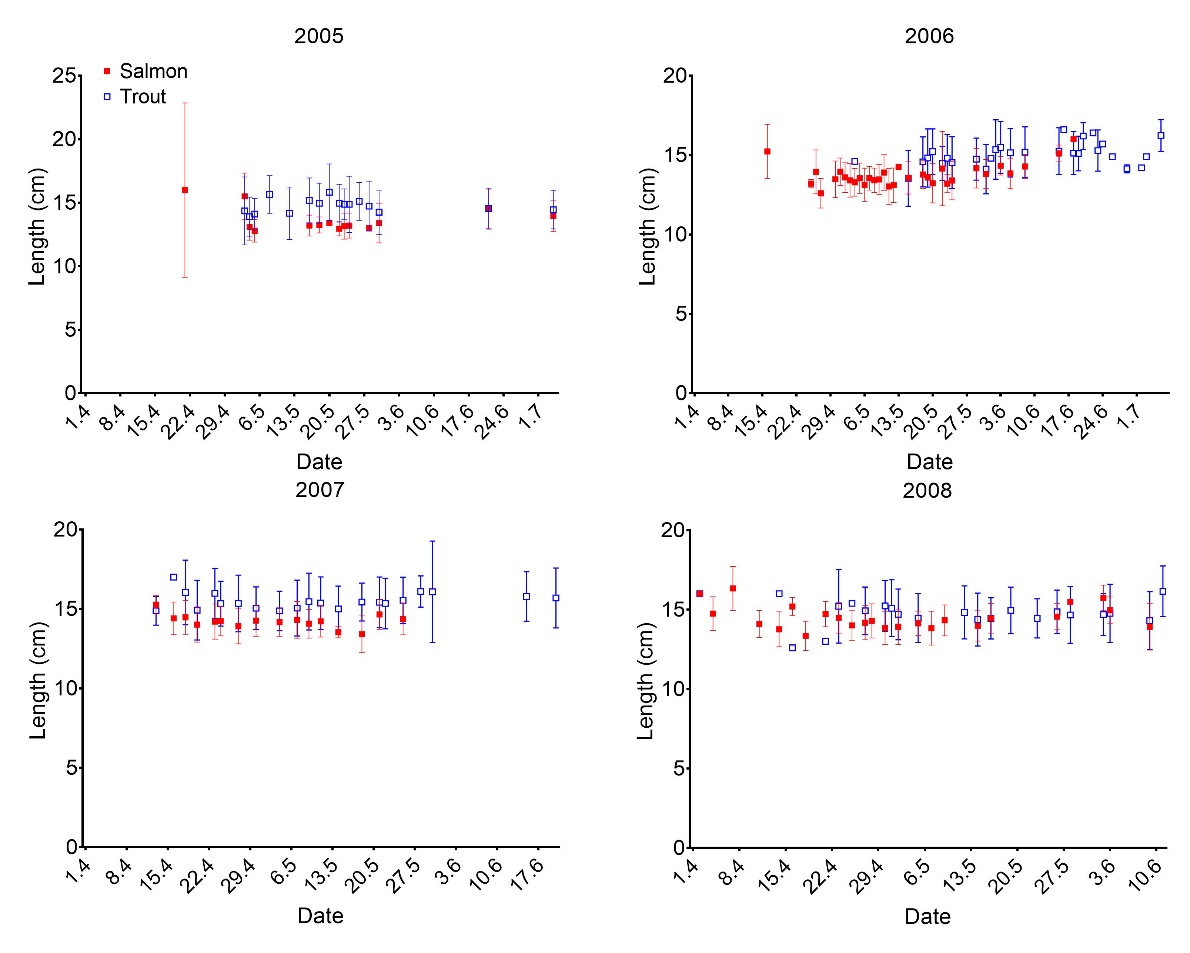


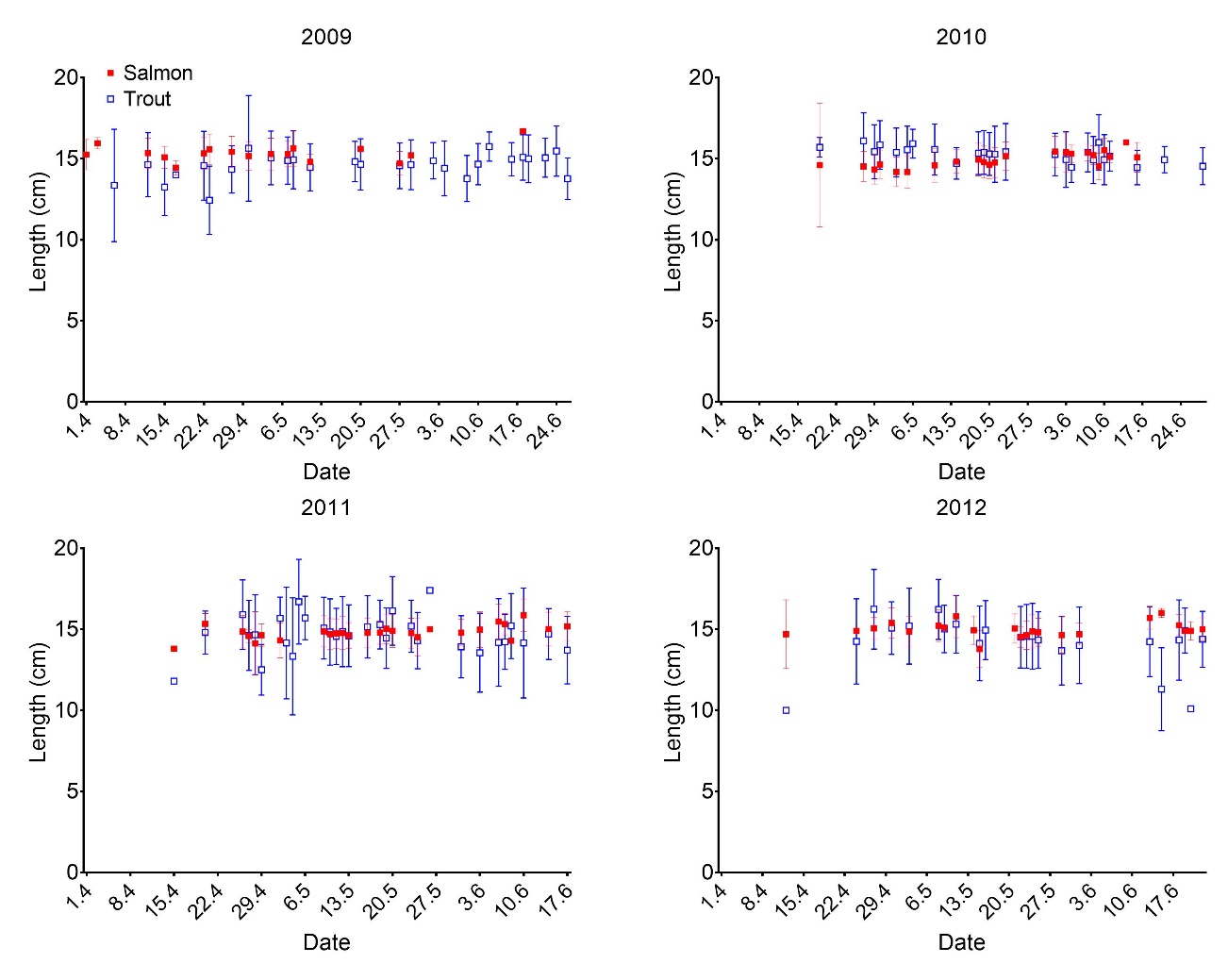

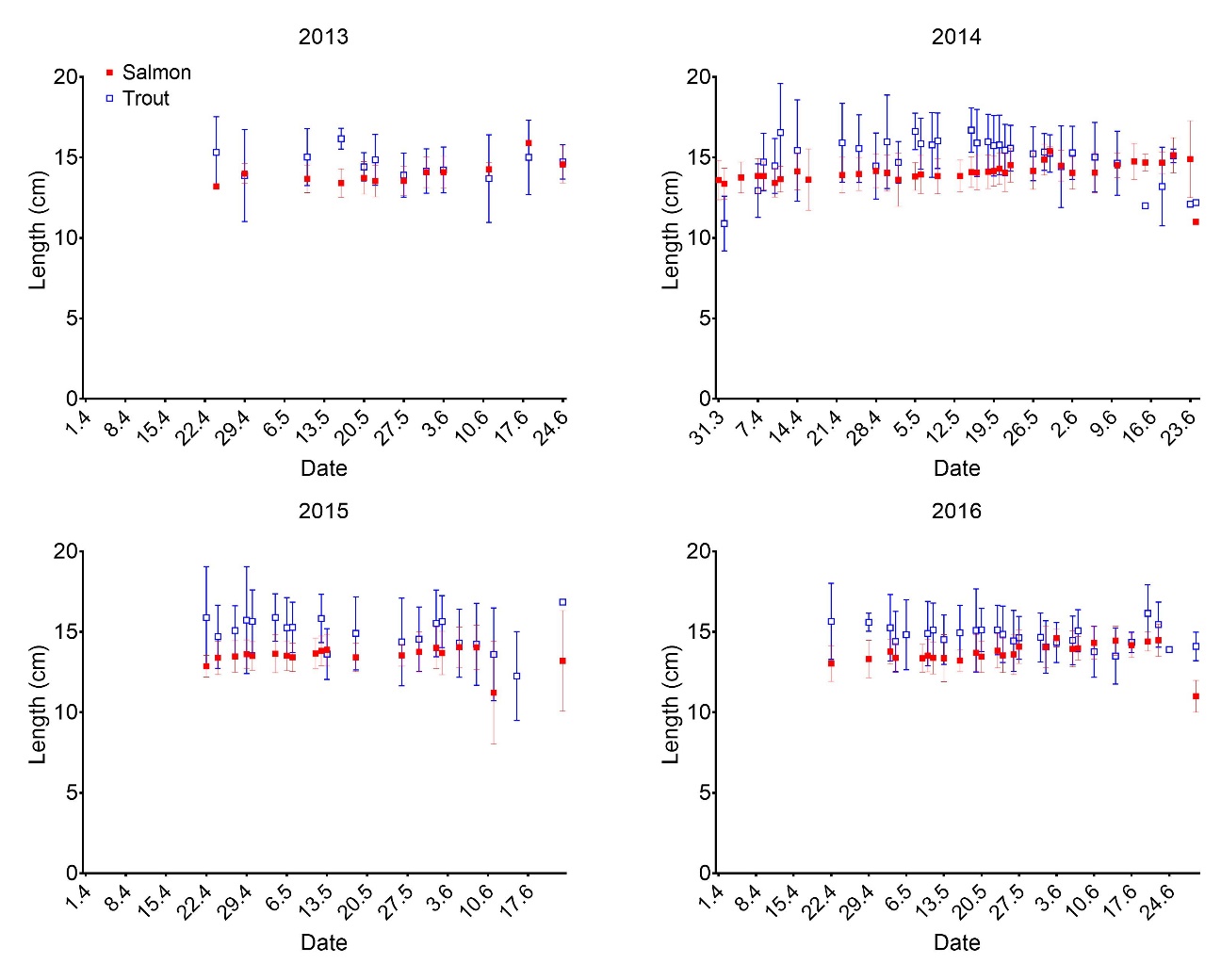


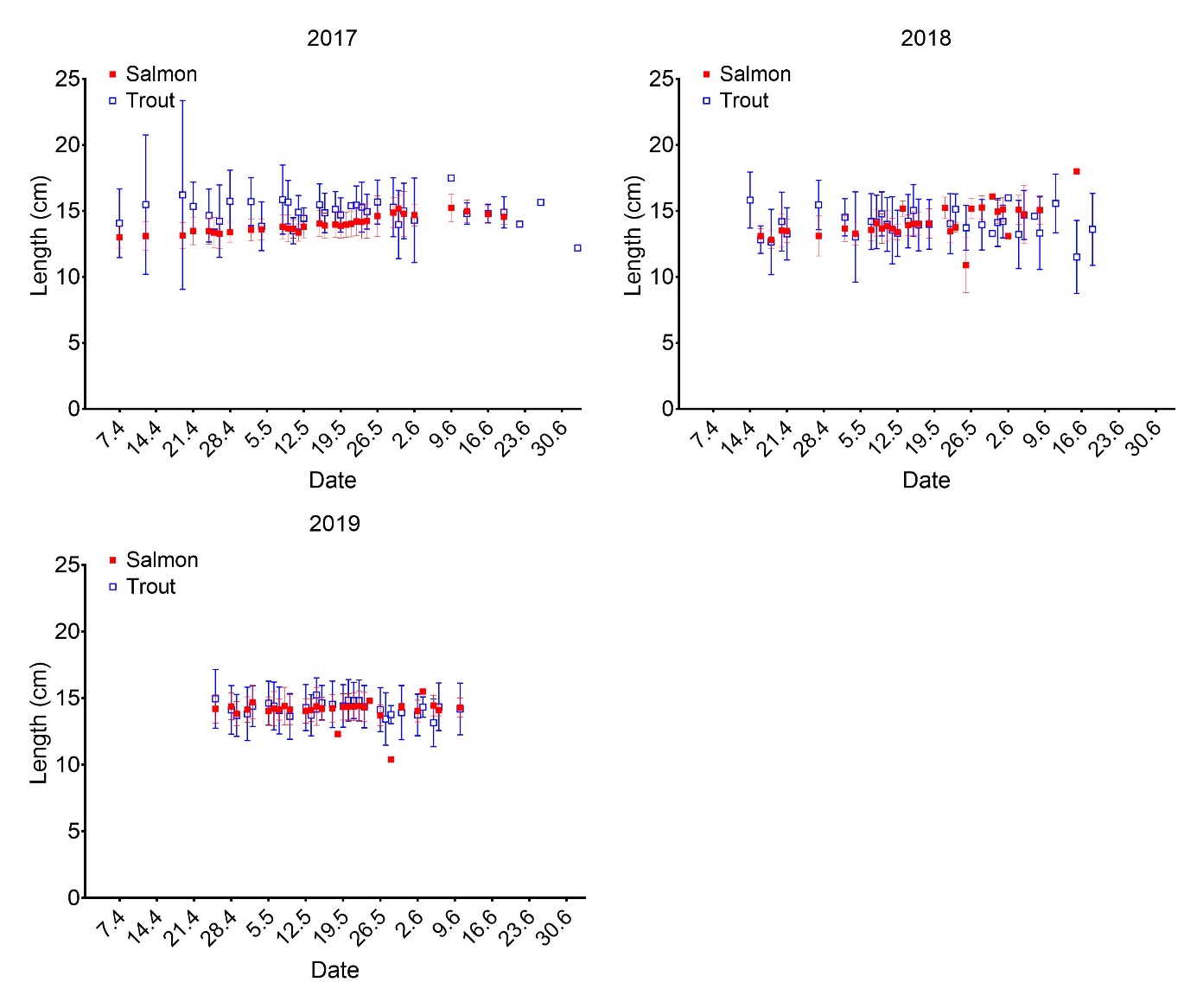


Figure S10: Average (± standard deviation) lengths (cm) of Atlantic salmon and sea trout migrating from the river per year and per day over the total study period (2001 – 2019).
